# Identification of stromal microenvironment characteristics and key molecular mining in pancreatic cancer

**DOI:** 10.1007/s12672-022-00532-y

**Published:** 2022-08-25

**Authors:** Hongchen Ji, Qiong Zhang, Xiang-Xu Wang, Junjie Li, Xiaowen Wang, Wei Pan, Zhuochao Zhang, Ben Ma, Hong-Mei Zhang

**Affiliations:** 1 Department of Oncology, Xijing Hospital, Fourth Military Medical University, No. 127 West Changle Road, Xi’an, 710032 China; 2 Department of Emergency, Xijing Hospital, Fourth Military Medical University, No. 127 West Changle Road, Xi’an, China; 3 Faculty of Hepatopancreatobiliary Surgery, Chinese PLA General Hospital, No. 28 Fuxing Road, Beijing, China

**Keywords:** Pancreatic cancer, Extracellular matrix, Metabolism, Immune microenvironment, Kelch like Family Member 32

## Abstract

**Purpose:**

Pancreatic cancer is one of the deadliest cancers worldwide. The extracellular matrix (ECM) microenvironment affects the drug sensitivity and prognosis of pancreatic cancer patients. This study constructed an 8-genes pancreatic ECM scoring (PECMS) model, to classify the ECM features of pancreatic cancer, analyze the impact of ECM features on survival and drug sensitivity, and mine key molecules that influence ECM features in pancreatic cancer.

**Methods:**

GSVA score calculation and clustering were performed in TCGA-PAAD patients. Lasso regression was used to construct the PECMS model. The association between PECMS and patient survival was analyzed and validated in the CPTAC-3 dataset of TCGA and our single-center retrospective cohort. The relationships between PECMS and features of the matrix microenvironment were analyzed. Finally, PECMS feature genes were screened and verified in pancreatic cancer specimens to select key genes associated with the ECM microenvironment.

**Result:**

The survival of the PECMS-high group was significantly worse. The PECMS-high group showed higher oxidative stress levels, lower levels of antigen presentation- and MHC-I molecule-related pathways, and less immune effector cell infiltration. Data from IMvigor-210 cohort suggested that PECMS-low group patients were more sensitive to immune checkpoint blockers. The PECMS score was negatively correlated with chemotherapy drug sensitivity. The negative association of PECMS with survival and drug sensitivity was validated in our retrospective cohort. KLHL32 expression predicted lower oxidative stress level and more immune cells infiltrate in pancreatic cancer.

**Conclusion:**

PECMS is an effective predictor of prognosis and drug sensitivity in pancreatic cancer patients. KLHL32 may play an important role in the construction of ECM, and the mechanism is worth further study.

**Supplementary Information:**

The online version contains supplementary material available at 10.1007/s12672-022-00532-y.

## Introduction

Pancreatic ductal adenocarcinoma (PDAC) is one of the leading causes of cancer-related death worldwide [1]. Pancreatic cancer causes 466,003 deaths, which is 4.7% of cancer-related deaths worldwide [2]. Patients with pancreatic cancer have a poor prognosis, with a 5-year overall survival (OS) rate of less than 10% [2]. Over 80% of patients have locally advanced or metastatic disease when they are first diagnosed and are not suitable for direct surgical treatment. Even in patients who undergo radical operations, most experience recurrence within 5 years [3]. Therefore, improving the effect of drug therapy is key to improving the survival of patients with pancreatic cancer.

Chemotherapy for pancreatic cancer is usually based on 5-fluorouracil (5-FU) or gemcitabine. In recent years, the use of drugs including albumin-bound paclitaxel and liposome irinotecan, as well as improvements in combination therapy strategies, have improved patient survival [4, 5]. However, in most clinical studies, chemotherapy improved survival by only a few months [5, 6]. New therapeutic strategies, such as angiogenesis inhibitors and immune checkpoint blockers (ICBs), have shown potential in preclinical studies [7, 8] but still face problems of poor effects or limited benefits in clinical trials [9]. Therefore, it is still a challenge to explore new systematic treatment strategies for pancreatic cancer.

In recent years, increasing attention has been given to the effects of the extracellular matrix (ECM) on survival and tumor biological behavior in pancreatic cancer. Pancreatic cancer is a dense tumor characterized by excessive connective tissue hyperplasia in histopathology [10–12]. In pancreatic cancer, the main components of the ECM include collagen, hyaluronic acid, and laminin [11, 13, 14]. Several studies have suggested a correlation between ECM features and the prognosis of pancreatic cancer patients. Whatcott et al. found that the median survival time of patients characterized by high hyaluronic acid levels in the ECM was 9.3 months, while patients characterized by low hyaluronic acid levels in the ECM had a significantly longer median survival of 24.3 months [15, 16]. In the same study, a high level of type I collagen in the ECM is also predictive of worse survival. However, when total collagen was investigated in grouping, no correlations with patient outcomes were found. This finding indicated that other components need to be taken into consideration in the analysis of the pancreatic cancer ECM. Another reason to focus on the ECM is that it affects the delivery of therapeutic drugs. The dense ECM in pancreatic cancer increases tissue tension, leads to difficulty in angiogenesis and intratumoural vessel collapse, and increases the difficulty of drug transport [17].

For these reasons, several drugs have been developed to target the ECM in pancreatic cancer [18–20]. However, although preclinical studies have shown promising prospects for treatments targeting the pancreatic cancer ECM, there is still no effective anti-ECM treatment. Anti-angiogenesis therapies, which normalize tumor vessels and improve drug delivery in pancreatic cancer, did not meet expectations in clinical trials [21, 22]. Other methods targeting the ECM of pancreatic cancer, such as PEGPH20, a PEGylated hyaluronidase targeting hyaluronic acid, were effective in preclinical studies [23] but did not inhibit tumor progression or improve survival in clinical trials [24]. The outcomes of clinical trials indicated the complexity of treatment targeting the pancreatic cancer ECM. An overview of the ECM of pancreatic cancer, the biological characteristics of different ECM types, and the key regulatory molecules of the ECM still need further study to explore new treatments targeting the ECM in pancreatic cancer.

## Methods and materials

### Data sources

The workflow of our study is shown in Fig. S1. The mRNA expression data, somatic mutation data, and clinical information, including age, sex, survival time, tumor stage, and histology type of TCGA-PAAD and CPTAC-3 were obtained from the TCGA database (https://portal.gdc.cancer.gov, RRID:SCR_014514). The data were analyzed using R (version 3.6.2) and R Bioconductor packages (RRID:SCR_006442).

We also collected data from a renal cancer cohort (IMvigor-210) using atezolizumab (and anti-PD-L1 antibody) for the validation of ICB effectiveness prediction [25]. The IMvigor-210 cohort with expression data and detailed clinical notes were downloaded from http://research-pub.gene.com/IMvigor210CoreBiologies. Expression and clinical information were downloaded from https://doi.org/10.5281/zenodo.

### GSVA scoring and hierarchical clustering

We used the "GSVA" package in R to acquire pathway scores to obtain clusters for patients in TCGA-PAAD and to study the relationship between transcription and biological process differences [26]. The hallmark gene set “h.all.v7.2” for GSVA was downloaded from the MSigDB database (https://www.gsea-msigdb.org/gsea/msigdb/). Hierarchical clustering was used to cluster TCGA-PAAD patients according to ECM-associated pathways.

### Construction of the PECMS system to evaluate the ECM of pancreatic cancer

By converting the read count data into log2 conversion, the “limma” R package estimates the mean variance relationship before linear modeling [28]. Empirical Bayesian statistics were used to analyze the differentially expressed genes (DEGs) between different RNA modification patterns. Genes with |log2 fold change (FC)|> 1.4 between groups and false discovery rate (FDR) < 0.0001 were regarded as having significant differences.

Lasso regression was used to select an optimal gene set [29]. The selected genes were then input into a logistic regression model. The regularization parameter (lambda) was estimated using tenfold cross-validation. After the coefficients corresponding to each gene, as well as the intercept value in logistic regression, were obtained, the PECMS score of each sample was defined as $\sum_{0}^{i}{(Coef}_{i} \times {Exp}_{i)})+Intercept$, in which i represents the feature gene in the selected gene set.

### Association analysis of PECMS and drug sensitivity

The "pRRophetic" R package was used to predict the response to chemotherapeutic and targeted drugs of patients. The sensitivity value predicted by this package was based on the half maximal inhibitory concentration (IC50) of each pancreatic cancer sample in the Genomics of Drug Sensitivity in Cancer (GDSC) database.

### Calculation of tumor microenvironment (TME) cell infiltration abundance

CIBERSORT (https://cibersort.stanford.edu/, RRID:SCR_016955) was used to calculate the infiltration abundance of 22 kinds of immune cells in TCGA-PAAD. The reference for the 22 kinds of immune cells referred to Newman et al. [30].

### Collection of clinical specimens and data

This study was approved by the Ethics Committee of the First Affiliated Hospital of AirForce Medical University (KY20223414-1). We initiated a retrospective study that included 20 patients who were diagnosed with PDAC in 2018 and underwent radical surgery in our hospital. All the patients were younger than 75 years old and were confirmed as PADC by pathology. The included patients did not receive adjuvant therapy due to poor Eastern Cooperative Oncology Group performance status score or postoperative complications. All the patients relapsed after surgery and received albumin-bound paclitaxel plus gemcitabine as first-line chemotherapy. Patients dead of postoperative complications were excluded. The collected clinical data included gender, age, tumor stage, disease-free survival (DFS), OS, and best response. All response evaluations followed the Response Evaluation Criteria in Solid Tumors (RECIST) 1.1 standard. Surgical specimens were collected for RNA sequencing and immunohistochemical (IHC) staining. Every patient signed the informed consent form. All patients were anonymized to meet ethical and legal standards.

### RNA sequencing

Total RNA was extracted using the Total RNA Extractor (Trizol) Kit (B511311, Sangon, China) according to the manufacturer’s protocol and treated with RNase-free DNase I to remove genomic DNA contamination. Thereafter, the quality and quantity of RNA were assessed using a NanoPhotometer® spectrophotometer (IMPLEN, CA, USA) and a Qubit^®^ 2.0 Fluorometer (Invitrogen). The high-quality RNA samples were subsequently submitted to Sangon Biotech (Shanghai) Co., Ltd. For library preparation and sequencing. Library quality was assessed on an Agilent Bioanalyzer 2100 system. The libraries were then quantified and pooled. Paired-end sequencing of the library was performed on NovaSeq sequencers (Illumina, San Diego, CA).

### IHC staining

The antibodies used in IHC staining include anti-CD8 (Abcam, ab237709), anti-CD31 (Abcam, ab28364, RRID: AB_2892677), anti-PD-L1 (proteintech, 66,248–1-Ig), and anti-GLUT1 (Abcam, ab115730). After dewaxing and rehydrating the slices, they were microwave heated for 15 min in 10 mM citric acid buffer (pH 6.0) to extract antigen. Then, 3% hydrogen peroxide was used to block endogenous peroxidase activity. The slices were then blocked with 10% goat serum for 2.5 h. After incubation with the primary antibodies or control IgG at 4 °C overnight, the slices were washed and incubated with a secondary antibody. After washing, the slices were incubated with biotin secondary antibody and the streptavidin–biotin complex for 30 min at room temperature. After washing with PBS, the substrate was then immersed in 0.4 mg/mL 3, 30-diaminobenzidine (DAB) with 0.003% hydrogen peroxide for 5 min. Finally, the slices were rinsed with distilled water, counterstained with hematoxylin, dehydrated, and then coverslipped.

### Statistical analysis

One‐way ANOVA was used for the group comparisons of continuous variables, and the χ2 test was used for that of categorical variables. Pearson's correlation test was used to analyze the correlations between mRNA expression and drug sensitivity. A receiver operating characteristic (ROC) curve was used to test the model validity, in which the area under the curve (AUC) was used as an evaluation index for the model. The "maftools" R package was used to obtain the mutation landscape and tumor mutational burden (TMB) value of each sample. In the mutation landscape [27]. Kaplan–Meier plots were generated using the R packages “survival” v.2.4.2 (40) and “survminer” v.0.4.2 (41). Samples with an OS of less than one month were omitted from survival analyses. Survival curves were constructed by the Kaplan–Meier method and compared by the log rank test. Univariate and multivariate Cox regression analyses were used to calculate the hazard ratio (HR) of different variables. A two‐sided P < 0.05 was regarded as statistically significant. Statistical analyses were carried out using the "scipy.stats" (RRID:SCR_008058) python package (RRID:SCR_001658) or R software.

## Result

### Identification of ECM subtypes in pancreatic cancer

**Fig. 1:**
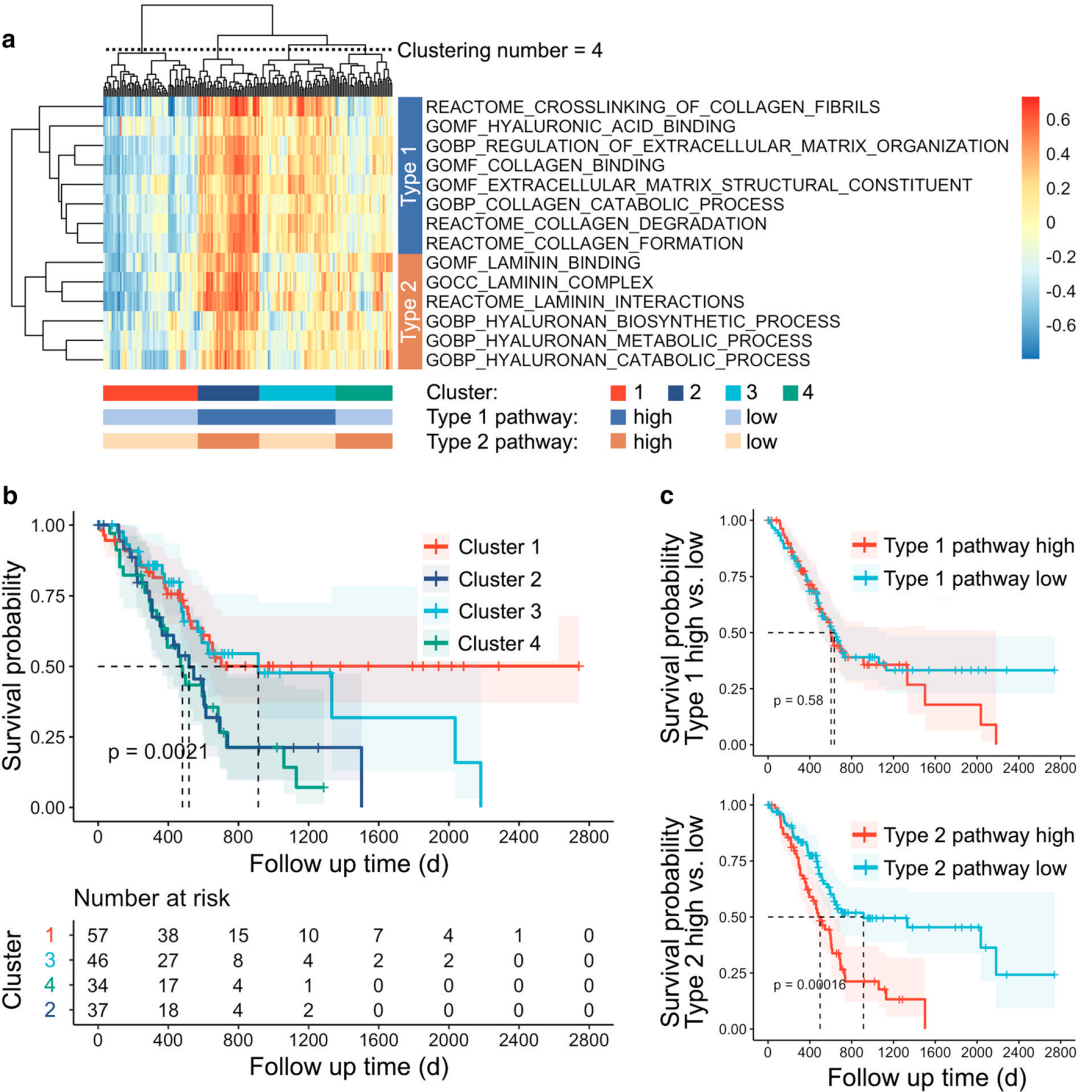
TCGA-PAAD patient clustering based on pancreatic cancer matrix (**a** Heatmap of GSVA hierarchical clustering of TCGA-PAAD. **b** Survival curves of 4 classes obtained by hierarchical clustering. **c** Survival curves of GSVA score high vs. low in Type 1 and 2 pathways, respectively.)

Fourteen pathways related to the pancreatic cancer ECM were selected from the Gene Ontology (GO) and REACTOME pathway databases, including the biological processes of collagen, hyaluronic acid, and laminin, as well as ECM organization, construction, and interaction with cancer cells. GSVA was performed in 174 patients in the TCGA-PAAD cohort to obtain a score for each pathway. By performing hierarchical clustering according to GSVA scores, we obtained 4 patient categories and 2 pathway categories (Fig. 1a). The Type 1 pathways include collagen synthesis, metabolism, binding, ECM construction, and binding of hyaluronic acid, while the Type 2 pathways mainly include the biological processes of hyaluronic acid and laminin. A significant difference in survival was observed among the four groups (P = 0.0021). Cluster 1 (n = 57, median survival = NR, 95% Cl 596-NR) and Cluster 3 (n = 46, median survival = 913, 95% Cl 568-NR) had better survival than Cluster 2 (n = 37, median survival = 518, 95% Cl 353–738) and Cluster 4 (n = 34, median survival = 418, 95% Cl 366–732) (Fig. 1b). The contribution of the GSVA score to survival in the Type 1 and Type 2 pathway categories was then analyzed. The results indicated that there was no significant difference in survival between patients with high and low scores in Type 1 pathways, while the GSVA score of Type 2 pathways was significantly correlated with survival (Fig. 1c).

### Construction of the pancreatic cancer ECM scoring model

**Fig. 2:**
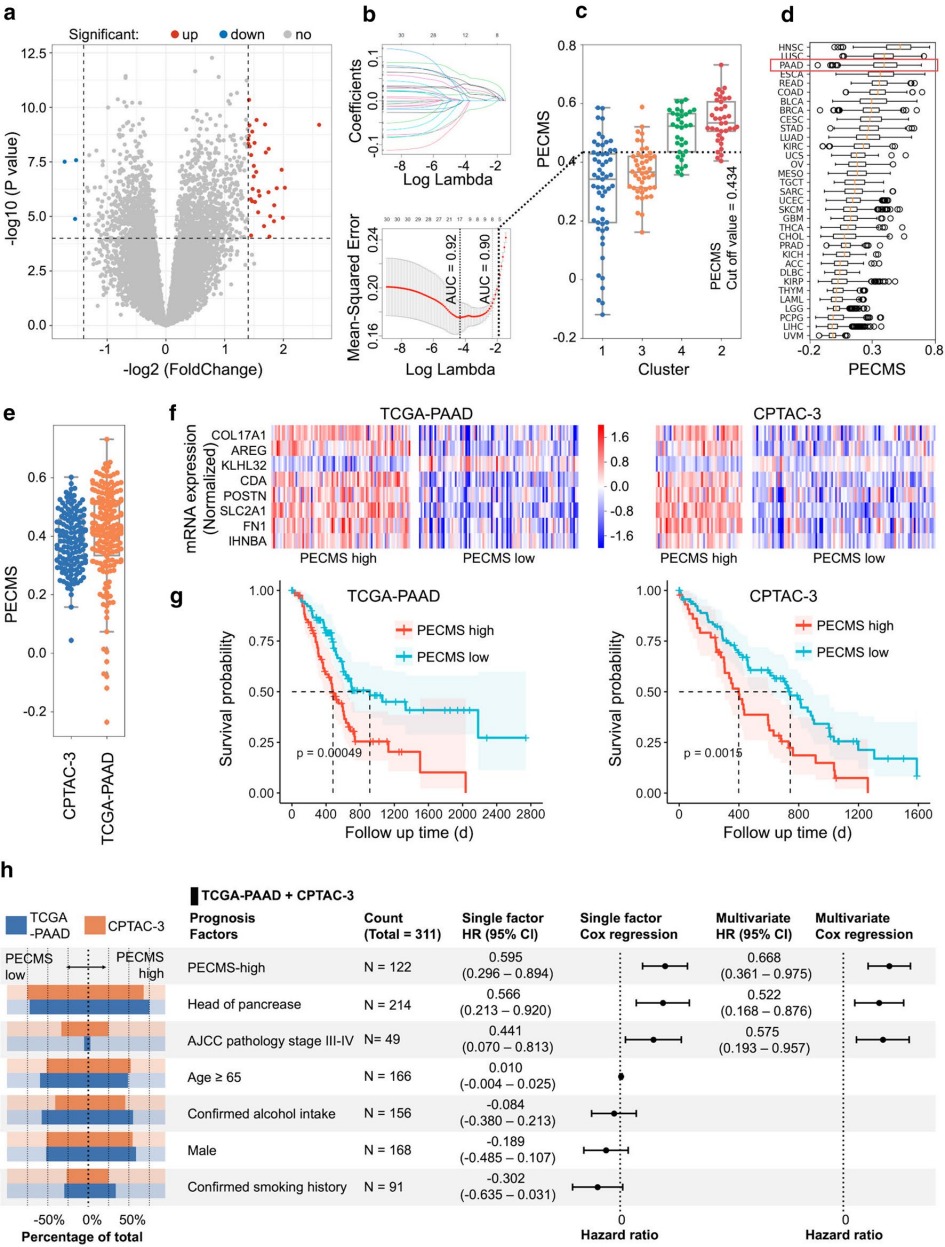
Establishment of the PECMS model and the effect of PECMS on survival (**a** Limma analysis to identify differentially expressed genes between the PECMS-high and PECMS-low groups. **b** Lasso process, AUCs of the model when the log lambda value at the minimum MSE and 1SE MSE were 0.90 and 0.92, respectively. **c** PECMS in different clusters of patients. **d** PECMS in different cancers in TCGA database. **e** PECMS in patients of the TCGA-PAAD and CPTAC-3 cohorts. **f** mRNA expression of PECMS feature genes in TCGA-PAAD and CPTAC-3. **g** Survival curves of TCGA-PAAD and CPTAC-3. Patients were grouped by the level of PECMS. **h** Clinical features, including PECMS in TCGA-PAAD and CPTAC-3, as well as their HR for survival in single-factor and Multivariate Cox analyses.)

The PECMS model was constructed based on the GSVA scores of the Type 2 pathways. Through limma analysis, we screened 31 genes significantly different at the transcriptional level between the Type 2 score-high group and the Type 2 score-low group (Fig. 2a, Supplementary Table S1). Based on these genes, a prediction model was constructed by Lasso regression. When the mean squared error (MSE) of Lasso regression was taken as the minimum value and when one standard error (1 SE) of the MSE was used, 17 and 8 genes were selected for model construction, respectively (Fig. 2b, Supplementary Table S2). The AUCs of the two sets of parameters were 0.93 and 0.90, respectively. Cutoff values for PECMS were decided according to the maximum Youden index (Supplementary Fig. S2). The two sets of parameters had similar AUCs and accuracy rates, but the scoring model based on 1SE MSE significantly reduced the number of variables and had a more balanced sample size between groups in the TCGA-PAAD cohort (81 in the PECMS-high group and 93 in the PECMS-low group) than the model based on the minimum MSE (64 in the PECMS-high group and 110 in the PECMS-low group). Based on the parameters obtained from the 1SE MSE of Lasso regression, a total of 8 genes (COL17A1, AREG, KLHL32, CDA, POSTN, SLC2A1, FN1, and INHBA) were included to obtain PECMS. The PECMS-high group contained many Cluster 2 and Cluster 4 patients, while the PECMS-low group contained a high proportion of Cluster 1 and Cluster 3 patients (Fig. 2c). Among the solid tumors included in TCGA database, pancreatic cancer ranked 3rd in PECMS and was the highest among all kind of adenocarcinomas (Fig. 2d). Another pancreatic cancer cohort in TCGA, CPTAC-3, was used to verify the above results. The PECMS scores of patients in the PCTAC-3 cohort were close to those of patients in the TCGA-PAAD cohort (Fig. 2e). Except for KLHL32, the remaining feature genes had higher transcriptional levels in the PECMS-high group, while the expression of KLHL32 showed the opposite trend (Fig. 2f). In both cohorts, the median survival of the patients in the PECMS-low group was significantly better than that of patients in the PECMS-high group (TCGA-PAAD: 913 d, 95% Cl 603-NR vs. 481 d, 95% Cl 381–634, P = 0.00049; CPTAC-3: 743 d, 95% Cl 599–902 vs. 398 d, 95% 303–603, P = 0.0015) (Fig. 2g). In the TCGA-PAAD and CPTAC-3 datasets, the clinical characteristics of gender, age, American Joint Committee on Cancer (AJCC) stage, smoking history and drinking history were similar between the two groups. The PECMS score, site of the primary lesion, and AJCC stage were prognostic risk factors in univariate Cox analysis combining the two datasets. In multivariate Cox regression analysis, the PECMS score, site of lesion, and tumor stage were independent risk factors for prognosis (Fig. 2h).

### PECMS was associated with ECM characteristics

**Fig. 3:**
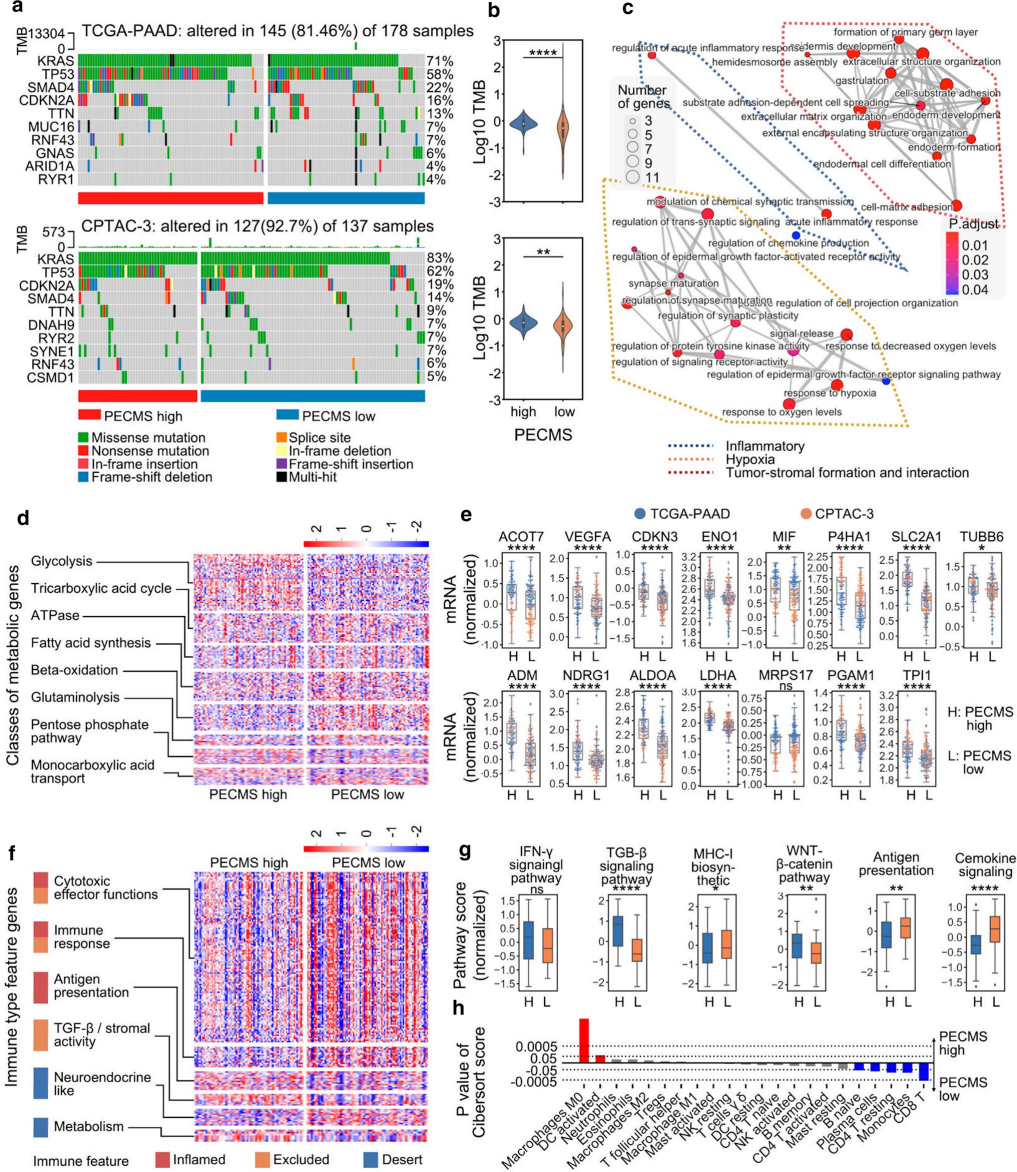
Microenvironment characteristics of patients in different PECMS classes (**a** Mutations in patients in different PECMS classes in TCGA-PAAD or CPTAC-3. **b** TMB of different PECMS classes in TCGA-PAAD or CPTAC-3. **c** Pathway pairwise enrichment between the PECMS-high and PECMS-low groups. d The normalized mRNA expression of metabolic features. **e** Different mRNA levels of 15 oxidative stress marker genes. **f** The normalized mRNA expression of immune feature genes.** g** The normalized pathway GSVA scores in different PECMS groups. **h** P value of CIBERSORT scores between different PECMS groups. ns: no significant difference; *: P < 0.05; **: P < 0.005; ***: P < 0.0005; ****: P < 0.00005)

In both the TCGA-PAAD and CPTAC-3 cohorts, KRAS, TP53, CDKN2A and SMAD4 had high mutation rates. The major mutation type of KRAS was missense mutations, and almost all PECMS-high patients had KRAS mutations, while the proportion of KRAS mutations in patients with PECMS-low was relatively low (Fig. 3a). In both cohorts, the TMB of patients in the PECMS-high group was significantly higher than that of patients in the PECMS-low group (Fig. 3b). In the differential pathway analysis based on the GO database, pathways were enriched in cell–matrix adhesion, ECM structure construction, collagen aggregation, and laminin complex (Supplementary Fig. S3). In addition to the ECM-related pathways used for clustering, two classes of pathways were enriched in pathway pairing analysis: the acute inflammatory response-related pathway and the hypoxia response-related and signal transduction pathway (Fig. 3c). Among the 156 representative genes of metabolic characteristics (Supplementary Table S3) [31], PECMS-high patients had higher transcriptional levels of glycolysis genes, while PECMS-low patients had higher transcriptional levels of fatty acid synthesis, β-oxidation and glutaminolysis genes (Fig. 3d). This suggests that there may be higher oxidative pressure in the pancreatic cancer tissues of PECMS-high patients. This result was verified in the analysis of the mRNA expression levels of oxidative stress marker genes [32]. Almost all these genes had significantly higher mRNA levels in the PECMS-high group (Fig. 3e). Interestingly, although there is a strong relationship between hypoxia and angiogenesis, no general significant differences were observed between the two groups in the expression of angiogenesis marker genes, including HIF-1α (HIF1A, a key gene for signaling in hypoxia) (Supplementary Fig. S4a). At the pathway level, there was also no significant difference in the GSVA score of angiogenesis between the two groups (Supplementary Fig. S4b). This suggests that the downstream signaling of hypoxia is at least partly blocked.

We then analyzed the immune characteristics of the different PECMS groups. In terms of the normalized GSVA scores, PECMS-low patients had higher scores in MHC-I biosynthesis and antigen presentation, which suggests a more active antigen recognition and presentation in these patients. Moreover, the chemokine signaling pathway, which plays an important role in immune cell infiltration and activation, was also upregulated in PECMS-low patients. PECMS-high patients had higher levels of the IFN-γ, TGF-β and WNT/β-catenin pathways (Fig. 3f, g; the gene markers [33] in Fig. 3f are listed in Supplementary Table S4). In CIBERSORT prediction of immune cell infiltration, the PECMS-low group had higher infiltration of immune effector cells, including significantly more CD8-positive T cells, activated CD4 T cells and activated NK cells. This finding agrees with the results of immune characteristics in the GSVA pathway analysis. The PECMS-high group had higher infiltration of M0 macrophages, activated DCs, etc. (Fig. 3h and Supplementary Fig. S5).

### PEMCS affect drug sensitive in pancreatic cancer

**Fig. 4:**
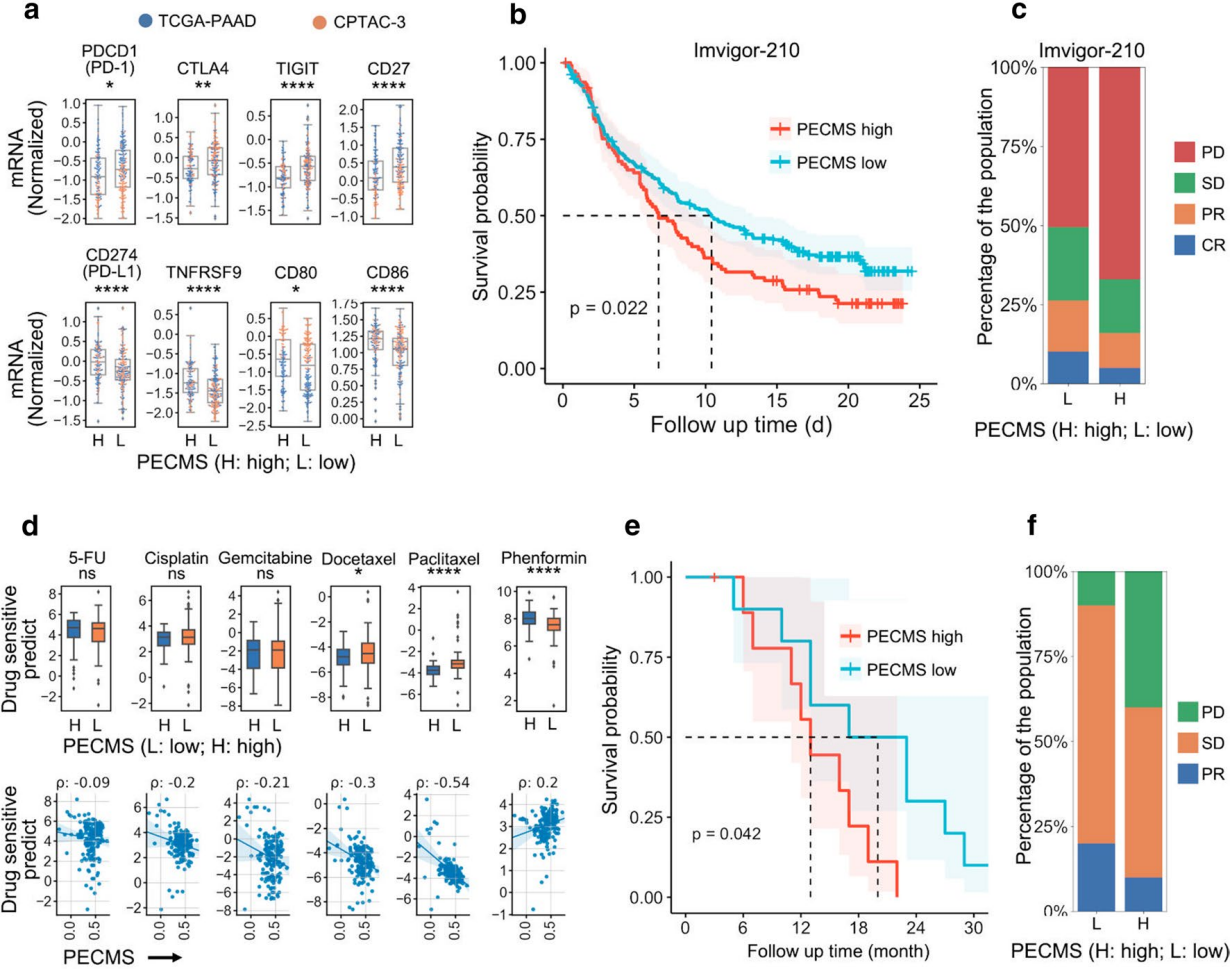
Relationship between PECMS and prediction of drug sensitivity (**a** The normalized mRNA expression of immune-privileged and ICB-sensitive biomarkers. **b** Survival of PECMS-high and PECMS-low patients in IMvigor-210. **c** Response of PECMS-high and PECMS-low patients in IMvigor-210. **d** Drug sensitivity prediction of chemotherapy drugs in different PECMS groups (L: PECMS-low; H: PECMS-high) and correlation between PECMS and drug sensitivity prediction. **e** Survival curves of different PECMS groups in our single-center retrospective cohort. **f** Response to chemotherapy in different PECMS groups in our single-center retrospective cohort. ns: no significant difference; *: P < 0.05; **: P < 0.005; ***: P < 0.0005; ****: P < 0.00005)

PECMS-high patients had higher transcription levels of immunotherapy predictors, including CD274 (PD-L1), TNFRSF9, CD80, and CD86. However, multiple immune depletion markers, including PDCD1 (PD-1), CTLA-4, TIGHT, and CD27, were significantly higher in the PECMS-low group (Fig. 4a), suggesting a high proportion of depleted T cells in the infiltrated immune cells of patients in the PECM-low group.

To further discuss the relationship between PECMS and the response to ICB therapy, we introduced an independent cohort (IMvigor-210) in which renal cell carcinoma patients were treated with ICB (atezolizumab, an anti-PD-L1 antibody). After aligning the mRNA data to TCGA-PAAD, 237 patients were divided into the PECMS-low group, and 111 patients were divided into the PECMS-high group. The median survival of the PECMS-low group was 10.4 months (95% Cl 8.02–13.31 months), which was significantly longer than that of the PECMS-high group (6.7 months, 95% Cl 5.65–9.49 months, P = 0.022) (Fig. 4b). A total of 198 patients in the PECMS-low group had response evaluation data. Twenty patients (10.1%) had the best outcome of complete response (CR), 32 (16.2%) had partial response (PR), 46 (23.2%) had stable disease (SD), and 100 (50.5%) had progressive disease (PD). In the 100 patients with response evaluation data in the PECMS-high group, the numbers of patients with the best efficacy of CR, PR, SD and PD were 5 (5%), 11 (11%), 46 (46%) and 67 (67%), respectively. Patients in the PECMS-low group had a significant response rate (P = 0.047) (Fig. 4c).

Among the main types of chemotherapeutic drugs approved for pancreatic cancer in the National Comprehensive Cancer Network (NCCN) guidelines, there was no significant difference between the PECMS-high and PECMS-low groups in the sensitivity to fluorouracil (5-FU or gemcitabine) and platinum. However, for taxane (paclitaxel and docetaxel), the PECMS-low group had a significantly higher sensitivity prediction. For all the selected chemotherapeutic drugs, PEMCS was negatively correlated with the drug sensitivity score. This partly explains the poor survival of patients in the PECMS-high group. The relationship between the PECMS score and sensitivity to phenformin (a biguanide hypoglycemic drug) was then analyzed. The results suggested that PECMS-high patients had a higher predicted sensitivity to phenformin, and PECMS showed a significant positive correlation with phenformin sensitivity (Fig. 4d).

Among the targeted medicines, we screened 17 drugs with significant differences in sensitivity prediction between the two groups. Seven drugs were predicted to be more efficient in the PECM-low group, including AICAR (AMPK agonist), MBS-754807 (IGF1R inhibitor), dasatinib (ABL inhibitor), thapsigargin (sarcoendoplasmic reticulum Ca2 + -ATPase inhibitor), and trametinib (MEK inhibitor), while CPT724714 (ERBB2 inhibitor), crizotinib (ALK inhibitor), GT-2580 (CSF1R inhibitor), masitinib (KIT inhibitor), OSI-027 (mTOR inhibitor), parthenolide (NFKB1 inhibitor), ruxolitinib (JAK inhibitor), shikonin (PKM2 inhibitor), and vorinostat (histone acetylase inhibitor) showed higher predictive sensitivity in the PECMS-high group (Supplementary Fig. S6).

### PECMS predicts survival in our retrospective cohort

In a retrospective cohort containing 20 patients from our single center, we further validated the associations of the PECMS score with survival and drug sensitivity (Supplementary Table S5). All patients were diagnosed with pancreatic cancer and underwent radical operation in 2018. The transcriptome data of these patients were obtained using surgical specimens. We transformed the transcriptome data of the included patients to have the same distribution as the data in TCGA-PAAD. PECMS scores were then calculated. We used the cut-off value consistent with TCGA-PAAD (0.434) to separate patients with PECMS-high and PECMS-low. According to the scoring results, 10 patients were included in the PECMS-high group, and 10 patients were included in the PECMS-low group. All patients received albumin paclitaxel and gemcitabine (AG) as first-line therapy after recurrence. The median OS of patients in the PECMS-low group (20 months, 95% Cl 13-NR) was significantly better than that of patients in the PECMS-high group (13 months, 95% Cl 11-NR, P = 0.042) (Fig. 4e). For the response to first-line AG therapy, 4 (40%) patients in the PECMS-high group had PD in the first-effect evaluation based on the RECIST 1.1 standard, while the number in the PECMS-low group was 1 (10%). Two patients in the PECMS-low group achieved PR; however, only 1 patient in the PECMS-high group achieved PR. The response to AG first-line therapy in the PECMS-low group was significantly better than that in the PECMS-high group (Fig. 4f). DFS of patients in the PECMS-low group (11 months, 95% Cl 7-NR) was also better than that of PECMS-high group (7 months, 95% Cl 5-NR), but the difference is not significant (P = 0.055) (Figure S8a). The results of immune features and drug sensitivity in the TCGA-PAAD dataset were also validated in our retrospective cohort, and the results of the 2 datasets showed similar characteristics. (Figure S8 b-d).

### KLHL32 is a key gene modeling the ECM of pancreatic cancer

**Fig. 5:**
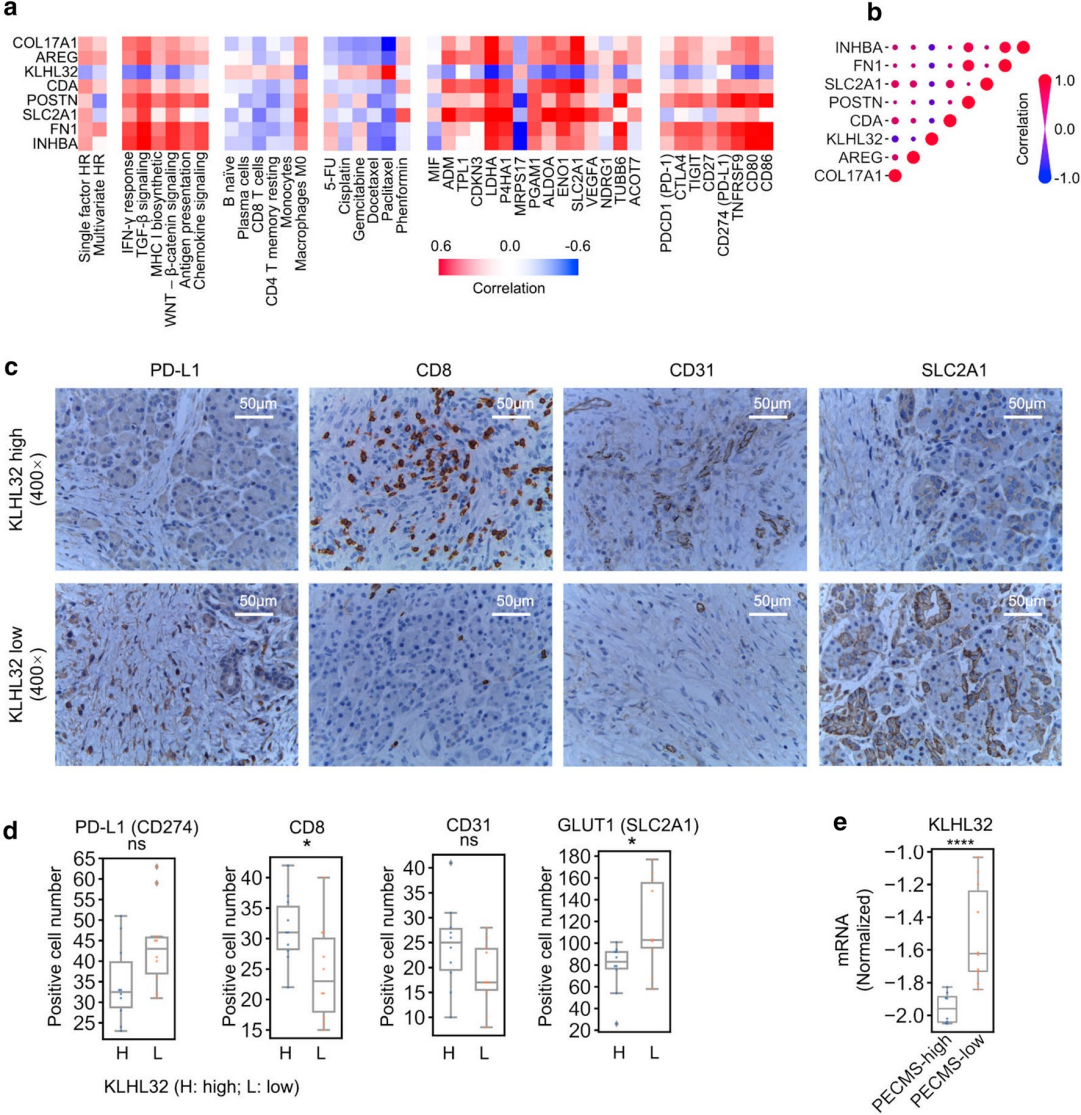
Relationship between KLHL32 and the pancreatic cancer ECM (**a** Correlation between PECMS feature genes and immune, metabolism, and drug sensitivity prediction characteristics. **b** Internal correlation among mRNA expression of PECMS feature genes. **c** Representative IHC images of PD-L1, CD8, CD31, and SLC2A1 in the KLHL32-high or KLHL32-low groups. **d** Comparison of mean positive cell number of IHC in 10 high power fields between the KLHL32-high (H) and the KLHL32-low (L) group. **e** mRNA of KLHL32 in PECMS-high and PECMS-low group. ns: no significant difference; *: P < 0.05)

We analyzed the correlation between eight molecules involved in the PECMS score and different clinical and tumor stromal features. KLHL32 showed distinct characteristics from the other molecules (Fig. 5a, b). In terms of survival, KLHL32 expression was associated with better prognosis in both univariate and multivariate Cox regression analyses, while COL17A1, AREG, FN1 and INHBA were associated with worse prognosis. We then set the median mRNA levels of each feature gene of PECMS as the cutoff value to divide the patients in the TCGA-PAAD cohort. In survival analysis, we found that for COL17A1, AREG, CDA, POSTN, SLC2A1, FN1 and INHBA, the median survival of the high expression group was significantly lower than that of the low expression group. However, for KLHL32, higher expression suggests better survival (Supplementary Fig. S7). This is consistent with the trend of univariate Cox analysis. Among the pathway correlations, KLHL32 showed the most obvious negative correlation with the TGF-β pathway. In drug sensitivity prediction, the expression of KLHL32 was positively correlated with the sensitivity to fluorouracil, platinum, and taxol. KLHL32 was negatively correlated with most of the oxidative stress markers, which was quite different from all the other molecular markers. In the CIBERSORT score of immune cell infiltration, KLHL32 was positively correlated with the infiltration of a variety of immune cells, including CD8-positive T cells, while it was negatively correlated with immune depletion markers (PDCD1, CTLA4, TIGHT, and CD27) (Fig. 5a).

In our retrospective cohort, the patients were divided into two groups using the median KLHL32 transcription level as the cutoff value. We then analyzed the relationship between KLHL32 levels and ECM phenotypes using GLUT-1 (SLC2A1) as a marker of oxidative stress and energy transport, CD31 as a marker of angiogenesis, CD8 as a marker of T-cell infiltration, and PD-L1 as a marker of immune escape. In the IHC staining of surgical specimens, low levels of CD31 and PD-L1 expression were observed in both groups. GLUT-1 expression was significantly lower in the KLHL32-high group than in the KLHL32-low group, deepening the relationship between KLHL32 and glucose transport in the ECM of pancreatic cancer and highlighting the role of GLUT1 in energy utilization in pancreatic cancer. Moreover, patients in the KLHL32-high group had more infiltrating CD8-positive cells, suggesting that KLHL32 plays a positive role in pancreatic cancer immune cell infiltration and the immune response (Fig. 5c, d, and Supplementary Fig. S9). Finally, we tested the KLHL32 mRNA level in PECMS-high and PECMS-low groups respectively. As shown in Fig. 5e, the KLHL32 level was significantly lower in the PECMS-high group, which is consistent with the results in TCGA and CPTAC-3.

## Discussion

An increasing number of studies are now focusing on the effects of the ECM on prognosis and drug sensitivity in pancreatic cancer, especially after the widespread use of antiangiogenic drugs and ICBs [34]. The sensitivity of these treatments is strongly correlated with the TME, including oxidative stress, angiogenesis, and immune cell infiltration [35]. In tissue specimens and enhanced scanning of CT or MR, pancreatic cancer is usually characterized by high density and a lack of blood supply [36]. Some studies have indicated that the high density of the ECM causes an increase in tissue tension and vascular collapse [17]. It is the main impediment for drug delivery and immune cell infiltration and ultimately reduces drug sensitivity in pancreatic cancer [37].

However, treatment targeting the ECM of pancreatic cancer has faced challenges in clinical research. In some studies, ECM-targeting medicines, such as PEGPH20, reduced survival. Studies on the ECM in pancreatic cancer have mainly focused on collagen and hyaluronic acid [23]. Some ECM components, such as collagen, are not only structural scaffolds but also bioactive materials in cancers, which have a direct impact on the biological behavior of tumor cells [16]. In addition, the construction of the ECM, as well as the interaction between cancer cells and the ECM, also play important roles in the ECM characteristics of pancreatic cancer [38]. These processes involve the regulation of multiple structural components in the ECM, including laminin, fibronectin, etc. Therefore, in this study, we included the biological processes of collagen, hyaluronic acid, and pathways related to ECM construction for clustering. When checking the GSVA score, it was observed that the levels of Type 1 and 2 pathways in the samples were not high or low. Many samples showed high or low expression in both Type 1 and Type 2 pathways. In the survival analysis, we analyzed the contributions of each pathway to survival and found that the Type 2 pathways were the main source of survival differences. Therefore, we constructed a pancreatic cancer classification scoring system (PECMS) using the Type 2 pathways. In the TCGA-PAAD, CPTAC-3, and our retrospective cohorts, PECMS-high patients had a worse prognosis, and PECMS was an independent risk factor for prognosis.

TMB is one of the widely acceptable effect biomarkers for ICBs [39]. Although TMB was higher in PECMS-high patients, it was at a low level in both groups. In fact, in the further validation in the IMvigor-210 cohort, the PECMS-high group had poor ICB treatment outcomes, suggesting that the relative level of TMB cannot predict ICB sensitivity in pancreatic cancer. We then analyzed the two enriched pathways related to ECM classification. There was an obvious difference in metabolic characteristics between the two groups, in which the transcription level of glycolysis-related genes was higher in the PECMS-high group, and the level of glutaminolysis marker genes was higher in the PECMS-low group. This suggested that the cancer tissues of PECM-high patients were exposed to higher oxidative stress, which was confirmed by the analysis of 15 oxidative stress marker genes. However, there was no significant difference in the levels of tricarboxylic acid cycling-related molecules between the two groups, suggesting that the PECMS-high group may have a higher glucose uptake level. It was reported that KRAS mutation can increase glucose transport by upregulating relevant genes [40, 41]. However, considering the high mutation rate of KRAS in both groups, the difference in glycometabolism and oxidative stress cannot be explained by the different mutation rates of KRAS.

SLC2A1 (GLUT1) is regarded as an energy-independent carbohydrate transporter and has a wide range of substrate properties [42]. SLC2A1 had a significant positive correlation with oxidative stress marker genes. In some studies, high expression of SLC2A1 is related to worse survival in pancreatic cancer [43, 44]. The high transcription level of SLC2A1 in the PECMS-high group suggests an energy-independent mode of carbohydrate transmembrane transport and energy utilization in the dense matrix of pancreatic cancer. Interestingly, although the transcription levels of almost all oxidative stress markers were higher in the PECMS-high group, there did not appear to be significant differences in most of the angiogenesis markers, particularly HIF1A (HIF-1α). HIF-1α is a key molecule that mediates angiogenesis under hypoxic stress [45]. This partly explained the lack of vessel formation in the ECM of pancreatic cancer, in which the inhibition of hypoxia signal downstream transduction may be one of the reasons.

In terms of immune characteristics, although broad evidence implicates IFN-γ in tumor immune surveillance, some reports suggested that it may also play a pro-tumorigenic role [46]. TGF beta also plays different roles in different stages of cancers. In the later stages, TGF-β is an immune suppression factor within the tumor microenvironment and leads to poor responses to cancer immunotherapy [47]. Moreover, TGF-β suppresses type 2 immunity to cancer [48]. Activation of the TGF-β pathway is considered one of the main causes of immune checkpoint inhibitor insensitivity. Mariathasan et al. [49] regarded TGF-β as one of the major reasons for the exclusion of T cells and led to the attenuation of the tumor response to ICBs. Meanwhile, because PECMS-high patients have lower levels of MHC-I, antigen presentation, and chemokine signaling pathways, PECMS-high seems to be related to a lower sensitivity to ICB treatment. This was verified in data from the IMvigor-210 cohort. These results suggest that targeting the ECM is a feasible strategy to enhance ICB sensitivity in pancreatic cancer. However, considering the different conditions of hemoperfusion and different immune infiltration statuses between renal cancer and pancreatic cancer, the predictive value of PECMS in ICB treatment still needs further study. In our drug sensitivity prediction of chemotherapeutic drugs, a high PECMS score usually predicts a worse response. This finding is consistent with the response rate data in our retrospective cohort. Due to the association between pancreatic cancer and glycolipid metabolism, biguanides, including metformin and phenformin, have long held the promise to improve the survival of pancreatic cancer [50]. However, the value of combination treatment with biguanides is still controversial in clinical trials [51]. In mechanistic studies, phenformin has been shown to suppress cancer progression by inhibiting epithelial-mesenchymal transformation and the ERK, AKT, or mTOR pathway [52]. In our study, phenformin showed a higher predictive sensitivity in the PECMS-high group, suggesting that the division of patients based on PECMS may help identify the potential benefits of biguanides.

Among the feature genes included in the PECMS score, we noticed the unique performance of KLHL32. It was a favorable factor for survival in both the single-factor and multivariable Cox regression analyses. A high negative correlation between KLHL32 and oxidative stress markers was observed, which suggested the role of KLHL32 in metabolic regulation [53, 54]. Moreover, KLHL32 is a positive factor of immune cell infiltration. KLHL32 belongs to the Kelch-like (KLHL) gene family. This gene family encodes proteins that constitute a subgroup at the intersection between the BTB/POZ domain and Kelch domain superfamilies [55–57]. Currently, there are very few studies on the function of KLHL32 in cancers. A few of studies have suggested an association between KLHL32 and BMI [50]. A GWAS study indicated that KLHL32 may be associated with obesity and type 2 diabetes [58]. These studies indirectly suggested a potential link between KLHL32 and glucolipid metabolism.

The condition of CD31 expression in the two groups confirmed that the hypoxic microenvironment in the pancreatic cancer matrix did not translate into the kinetic energy of angiogenesis, and the role of KLHL32 in blocking angiogenesis still needs further investigation. In our study, KLHL32 played a positive role in immune cell infiltration. However, the KLHL32 level is negatively correlated with ICB sensitivity predictors, such as PD-L1 and CTLA4. Hence, the relationship between KLHL32 and ICB sensitivity needs further study. In our PECMS model, KLHL32 appears to play a role in pulling the pancreatic cancer ECM in a positive direction for prognosis. Since the KLHL protein family regulates transcription by locally regulating chromatin conformation, the role of KLHL32 in transcriptional regulation may be extensive. Considering the unsatisfactory results of treatments targeting the ECM in pancreatic cancer, the role of KLHL32 in shaping the ECM and its value for drug development warrant more mechanistic and preclinical studies.

## Supplementary Information

**Supplementary file 1** (DOCX 6151 KB) **Fig. S1** Flowchart of this study. **Fig. S2** ROC curve of the Lasso model (Coefficient 1 was obtained at the minimum MSE, and coefficient 2 was obtained at 1 SE MSE. Cutoff values were obtained based on the Youden index). **Fig. S3** GO enrichment analysis between PECMS groups. **Fig. S4** Level of angiogenesis marker genes and pathway GSVA scores between PECMS groups (ns: no significant difference; *: P<0.05; **: P<0.005; ***: P<0.0005; ****: P<0.00005). **Fig. S5** CIBERSORT score value of different infiltrating immune cells between PECMS groups. **Fig. S6** Predictive value of molecular targeted drugs between PECMS groups (ns: no significant difference; *: P<0.05; **: P<0.005; ***: P<0.0005; ****: P<0.00005). **Fig. S7** Survival curve of TCGA-PAAD. The patients were divided by the median value of PECMS feature gene mRNA levels. **Fig. S8** Validation of disease-free survival, immunological characteristics, and drug sensitivity in our retrospective data set (a: Disease-free survival of different PECMS groups. b: The normalized mRNA expression of immune feature genes. c: The normalized pathway GSVA scores in different PECMS groups. d: Drug sensitivity prediction of chemotherapy drugs in different PECMS groups (L: PECMS-low; H: PECMS-high) and correlation between PECMS and drug sensitivity prediction). **Fig. S9** IHC of PD-L1, CD8, CD31, and GLUT1 (SLC2A1) in our single-center retrospective cohort (a: IHC staining, the patients were grouped by the level of KLHL32. b: Positive and negative controls of the 4 antibodies. CD31: human lung tissue; GLUT1: rat liver tissue; CD8: rat spleen tissue; PD-L1: rat heart tissue)

**Supplementary file 2** (XLSX 21 KB). **Table S1** Results of the limma analysis of significantly differentially expressed genes between the high and low Type 2 pathways. **Table S2** Coefficients of the PEMCS model. **Table S3** List of metabolism marker genes. **Table S4** List of immune feature marker genes. **Table S5** Clinical data and PECMS results of our single-center retrospective cohort of pancreatic cancer

## Data Availability

The data generated in this study are publicly available in Sequence Read Archive (SRA) with the id 11,196,981, and within the article and its supplementary data files. The raw data in SRA database is available for download after May 15, 2022. The mRNA expression data, somatic mutation data, and clinical information, including age, sex, survival time, tumor stage, and histology type of TCGA-PAAD and CPTAC-3 were obtained from the TCGA database (https://portal.gdc.cancer.gov/repository). The IMvigor-210 cohort with expression data and detailed clinical notes were downloaded from http://research-pub.gene.com/IMvigor210CoreBiologies. Expression and clinical information were downloaded from https://doi.org/10.5281/zenodo. The code used for data analysis are written in Python and R. All the code are available for download in GitHub at https://github.com/FreudDolce/PECMSCODES/.

## References

[1] Siegel RL, Miller KD, Fuchs HE, Jemal A (2021). Cancer statistics, 2021. CA Cancer J Clin.

[2] Sung H, Ferlay J, Siegel RL, Laversanne M, Soerjomataram I, Jemal A, Bray F (2021). Global Cancer Statistics 2020: GLOBOCAN Estimates of Incidence and Mortality Worldwide for 36 Cancers in 185 Countries. CA Cancer J Clin.

[3] De La Cruz MS, Young AP, Ruffin MT (2014). Diagnosis and management of pancreatic cancer. Am Fam Physician.

[4] McGuigan A, Kelly P, Turkington RC, Jones C, Coleman HG, McCain RS (2018). Pancreatic cancer: a review of clinical diagnosis, epidemiology, treatment and outcomes. World J Gastroenterol.

[5] Mizrahi JD, Surana R, Valle JW, Shroff RT (2020). Pancreatic cancer. Lancet.

[6] Von Hoff DD, Ervin T, Arena FP, Chiorean EG, Infante J, Moore M, Seay T, Tjulandin SA, Ma WW, Saleh MN, Harris M, Reni M, Dowden S, Laheru D, Bahary N, Ramanathan RK, Tabernero J, Hidalgo M, Goldstein D, Van Cutsem E, Wei X, Iglesias J, Renschler MF (2013). Increased survival in pancreatic cancer with nab-paclitaxel plus gemcitabine. N Engl J Med.

[7] Yamamoto K, Venida A, Yano J, Biancur DE, Kakiuchi M, Gupta S, Sohn ASW, Mukhopadhyay S, Lin EY, Parker SJ, Banh RS, Paulo JA, Wen KW, Debnath J, Kim GE, Mancias JD, Fearon DT, Perera RM, Kimmelman AC (2020). Autophagy promotes immune evasion of pancreatic cancer by degrading MHC-I. Nature.

[8] Moral JA, Leung J, Rojas LA, Ruan J, Zhao J, Sethna Z, Ramnarain A, Gasmi B, Gururajan M, Redmond D, Askan G, Bhanot U, Elyada E, Park Y, Tuveson DA, Gönen M, Leach SD, Wolchok JD, DeMatteo RP, Merghoub T, Balachandran VP (2020). ILC2s amplify PD-1 blockade by activating tissue-specific cancer immunity. Nature.

[9] Marabelle A, Le DT, Ascierto PA, Di Giacomo AM, De Jesus-Acosta A, Delord JP, Geva R, Gottfried M, Penel N, Hansen AR, Piha-Paul SA, Doi T, Gao B, Chung HC, Lopez-Martin J, Bang YJ, Frommer RS, Shah M, Ghori R, Joe AK, Pruitt SK, Diaz LA (2020). Efficacy of pembrolizumab in patients with noncolorectal high microsatellite instability/mismatch repair-deficient cancer results from the phase II KEYNOTE-158 study. J Clin Oncol..

[10] Armstrong T, Packham G, Murphy LB, Bateman AC, Conti JA, Fine DR, Johnson CD, Benyon RC, Iredale JP (2004). Type I collagen promotes the malignant phenotype of pancreatic ductal adenocarcinoma. Clin Cancer Res.

[11] Bachem MG, Schünemann M, Ramadani M, Siech M, Beger H, Buck A, Zhou S, Schmid-Kotsas A, Adler G (2005). Pancreatic carcinoma cells induce fibrosis by stimulating proliferation and matrix synthesis of stellate cells. Gastroenterology.

[12] Apte MV, Park S, Phillips PA, Santucci N, Goldstein D, Kumar RK, Ramm GA, Buchler M, Friess H, McCarroll JA, Keogh G, Merrett N, Pirola R, Wilson JS (2004). Desmoplastic reaction in pancreatic cancer: role of pancreatic stellate cells. Pancreas.

[13] Bonnans C, Chou J, Werb Z (2014). Remodelling the extracellular matrix in development and disease. Nat Rev Mol Cell Biol.

[14] Linder S, Castaños-Velez E, von Rosen A, Biberfeld P (2001). Immunohistochemical expression of extracellular matrix proteins and adhesion molecules in pancreatic carcinoma. Hepatogastroenterology.

[15] Whatcott CJ, Diep CH, Jiang P, Watanabe A, LoBello J, Sima C, Hostetter G, Shepard HM, Von Hoff DD, Han H (2015). Desmoplasia in primary tumors and metastatic lesions of pancreatic cancer. Clin Cancer Res.

[16] Öhlund D, Franklin O, Lundberg E, Lundin C, Sund M (2013). Type IV collagen stimulates pancreatic cancer cell proliferation, migration, and inhibits apoptosis through an autocrine loop. BMC Cancer.

[17] Di Maggio F, Arumugam P, Delvecchio FR, Batista S, Lechertier T, Hodivala-Dilke K, Kocher HM (2016). Pancreatic stellate cells regulate blood vessel density in the stroma of pancreatic ductal adenocarcinoma. Pancreatology.

[18] Zinger A, Koren L, Adir O, Poley M, Alyan M, Yaari Z, Noor N, Krinsky N, Simon A, Gibori H (2019). Collagenase nanoparticles enhance the penetration of drugs into pancreatic tumors. ACS Nano.

[19] Winer A, Adams S, Mignatti P (2018). Matrix metalloproteinase inhibitors in cancer therapy: turning past failures into future successes. Mol Cancer Ther.

[20] Ferrara B, Pignatelli C, Cossutta M, Citro A, Courty J, Piemonti L (2021). The extracellular matrix in pancreatic cancer: description of a complex network and promising therapeutic options. Cancers (Basel)..

[21] Rougier P, Riess H, Manges R, Karasek P, Humblet Y, Barone C, Santoro A, Assadourian S, Hatteville L, Philip PA (2013). Randomised, placebo-controlled, double-blind, parallel-group phase III study evaluating aflibercept in patients receiving first-line treatment with gemcitabine for metastatic pancreatic cancer. Eur J Cancer.

[22] Kindler HL, Ioka T, Richel DJ, Bennouna J, Létourneau R, Okusaka T, Funakoshi A, Furuse J, Park YS, Ohkawa S, Springett GM, Wasan HS, Trask PC, Bycott P, Ricart AD, Kim S, Van Cutsem E (2011). Axitinib plus gemcitabine versus placebo plus gemcitabine in patients with advanced pancreatic adenocarcinoma: a double-blind randomised phase 3 study. Lancet Oncol.

[23] Thompson CB, Shepard HM, O'Connor PM, Kadhim S, Jiang P, Osgood RJ, Bookbinder LH, Li X, Sugarman BJ, Connor RJ, Nadjsombati S, Frost GI (2010). Enzymatic depletion of tumor hyaluronan induces antitumor responses in preclinical animal models. Mol Cancer Ther.

[24] Ramanathan RK, McDonough SL, Philip PA, Hingorani SR, Lacy J, Kortmansky JS, Thumar J, Chiorean EG, Shields AF, Behl D, Mehan PT, Gaur R, Seery T, Guthrie KA, Hochster HS (2019). Phase IB/II randomized study of FOLFIRINOX plus pegylated recombinant human hyaluronidase versus FOLFIRINOX alone in patients with metastatic pancreatic adenocarcinoma: SWOG S1313. J Clin Oncol.

[25] Hoffman-Censits JH, Grivas P, Heijden MSVD, Dreicer R, Loriot Y, Retz M, Vogelzang NJ, Perez-Gracia JL, Rezazadeh A, Bracarda S, Yu EY, Hoimes CJ, Bellmunt J, Quinn DI, Petrylak DP, Hussain Syed A, Cui Na, Mariathasan S, Abidoye OO, Rosenberg JE (2016). IMvigor 210, a phase II trial of atezolizumab (MPDL3280A) in platinum-treated locally advanced or metastatic urothelial carcinoma (mUC). J Clin Oncol.

[26] Hänzelmann S, Castelo R, Guinney J (2013). GSVA: gene set variation analysis for microarray and RNA-seq data. BMC Bioinformatics.

[27] Mayakonda A, Lin DC, Assenov Y, Plass C, Koeffler HP (2018). Maftools: efficient and comprehensive analysis of somatic variants in cancer. Genome Res.

[28] Ritchie ME, Phipson B, Wu D, Hu Y, Law CW, Shi W, Smyth GK (2015). limma powers differential expression analyses for RNA-sequencing and microarray studies. Nucleic Acids Res.

[29] Tibshirani R (1996). Regression shrinkage and selection via the lasso. J Royal Statist Soc B.

[30] Newman AM, Steen CB, Liu CL, Gentles AJ, Chaudhuri AA, Scherer F, Khodadoust MS, Esfahani MS, Luca BA, Steiner D, Diehn M, Alizadeh AA (2019). Determining cell type abundance and expression from bulk tissues with digital cytometry. Nat Biotechnol.

[31] Prusinkiewicz MA, Gameiro SF, Ghasemi F, Dodge MJ, Zeng PYF, Maekebay H, Barrett JW, Nichols AC, Mymryk JS (2020). Survival-associated metabolic genes in human papillomavirus-positive head and neck cancers. Cancers (Basel).

[32] Mo Z, Liu D, Rong D, Zhang S (2021). Hypoxic characteristic in the immunosuppressive microenvironment of hepatocellular carcinoma. Front Immunol.

[33] Zhang B, Wu Q, Li B, Wang D, Wang L, Zhou YL (2020). m6A regulator-mediated methylation modification patterns and tumor microenvironment infiltration characterization in gastric cancer. Mol Cancer.

[34] Hosein AN, Brekken RA, Maitra A (2020). Pancreatic cancer stroma: an update on therapeutic targeting strategies. Nat Rev Gastroenterol Hepatol.

[35] Hessmann E, Buchholz SM, Demir IE, Singh SK, Gress TM, Ellenrieder V, Neesse A (2020). Microenvironmental determinants of pancreatic cancer. Physiol Rev.

[36] Vickman RE, Faget DV, Beachy P, Beebe D, Bhowmick NA, Cukierman E, Deng WM, Granneman JG, Hildesheim J, Kalluri R, Lau KS, Lengyel E, Lundeberg J, Moscat J, Nelson PS, Pietras K, Politi K, Puré E, Scherz-Shouval R, Sherman MH, Tuveson D, Weeraratna AT, White RM, Wong MH, Woodhouse EC, Zheng Y, Hayward SW, Stewart SA (2020). Deconstructing tumor heterogeneity: the stromal perspective. Oncotarget.

[37] Vennin C, Chin VT, Warren SC, Lucas MC, Herrmann D, Magenau A, Melenec P, Walters SN, Del Monte-Nieto G, Conway JR, Nobis M, Allam AH, McCloy RA, Currey N, Pinese M, Boulghourjian A, Zaratzian A, Adam AA, Heu C, Nagrial AM, Chou A, Steinmann A, Drury A, Froio D, Giry-Laterriere M, Harris NL, Phan T, Jain R, Weninger W, McGhee EJ, Whan R, Johns AL, Samra JS, Chantrill L, Gill AJ, Kohonen-Corish M, Harvey RP, Biankin AV, Evans TR, Anderson KI, Grey ST, Ormandy CJ, Gallego-Ortega D, Wang Y, Samuel MS, Sansom OJ, Burgess A, Cox TR, Morton JP, Pajic M, Timpson P, Australian Pancreatic Cancer Genome Initiative (APGI) (2017). Transient tissue priming via ROCK inhibition uncouples pancreatic cancer progression, sensitivity to chemotherapy, and metastasis. Sci Transl Med.

[38] Thomas D, Radhakrishnan P (2019). Tumor-stromal crosstalk in pancreatic cancer and tissue fibrosis. Mol Cancer.

[39] Luchini C, Bibeau F, Ligtenberg MJL, Singh N, Nottegar A, Bosse T, Miller R, Riaz N, Douillard JY, Andre F, Scarpa A (2019). ESMO recommendations on microsatellite instability testing for immunotherapy in cancer, and its relationship with PD-1/PD-L1 expression and tumour mutational burden: a systematic review-based approach. Ann Oncol.

[40] Zhang Q, Jeppesen DK, Higginbotham JN, Demory Beckler M, Poulin EJ, Walsh AJ, Skala MC, McKinley ET, Manning HC, Hight MR, Schulte ML, Watt KR, Ayers GD, Wolf MM, Andrejeva G, Rathmell JC, Franklin JL, Coffey RJ (2018). Mutant KRAS exosomes alter the metabolic state of recipient colonic epithelial cells. Cell Mol Gastroenterol Hepatol.

[41] Cenigaonandia-Campillo A, Serna-Blasco R, Gómez-Ocabo L, Solanes-Casado S, Baños-Herraiz N, Puerto-Nevado LD, Cañas JA, Aceñero MJ, García-Foncillas J, Aguilera Ó (2021). Vitamin C activates pyruvate dehydrogenase (PDH) targeting the mitochondrial tricarboxylic acid (TCA) cycle in hypoxic KRAS mutant colon cancer. Theranostics.

[42] Schüler R, Seebeck N, Osterhoff MA, Witte V, Flöel A, Busjahn A, Jais A, Brüning JC, Frahnow T, Kabisch S, Pivovarova O, Hornemann S, Kruse M, Pfeiffer AFH (2018). VEGF and GLUT1 are highly heritable, inversely correlated and affected by dietary fat intake: consequences for cognitive function in humans. Mol Metab.

[43] Chen X, Lu P, Zhou S, Zhang L, Zhao JH, Tang JH (2017). Predictive value of glucose transporter-1 and glucose transporter-3 for survival of cancer patients: a meta-analysis. Oncotarget.

[44] Lu K, Yang J, Li DC, He SB, Zhu DM, Zhang LF, Zhang XU, Chen XC, Zhang B, Zhou J (2016). Expression and clinical significance of glucose transporter-1 in pancreatic cancer. Oncol Lett.

[45] Jiang L, Li Y, He Y, Wei D, Yan L, Wen H (2021). Knockdown of m6A Reader IGF2BP3 inhibited hypoxia-induced cell migration and angiogenesis by regulating hypoxia inducible factor-1α in stomach cancer. Front Oncol.

[46] Castro F, Cardoso AP, Gonçalves RM, Serre K, Oliveira MJ (2018). Interferon-gamma at the crossroads of tumor immune surveillance or evasion. Front Immunol.

[47] Batlle E, Massagué J (2019). Transforming growth factor-β signaling in immunity and cancer. Immunity.

[48] Liu M, Kuo F, Capistrano KJ, Kang D, Nixon BG, Shi W, Chou C, Do MH, Stamatiades EG, Gao S, Li S, Chen Y, Hsieh JJ, Hakimi AA, Taniuchi I, Chan TA, Li MO (2020). TGF-β suppresses type 2 immunity to cancer. Nature.

[49] Mariathasan S, Turley SJ, Nickles D, Castiglioni A, Yuen K, Wang Y, Kadel EE, Koeppen H, Astarita JL, Cubas R, Jhunjhunwala S, Banchereau R, Yang Y, Guan Y, Chalouni C, Ziai J, Şenbabaoğlu Y, Santoro S, Sheinson D, Hung J, Giltnane JM, Pierce AA, Mesh K, Lianoglou S, Riegler J, Carano RAD, Eriksson P, Höglund M, Somarriba L, Halligan DL, van der Heijden MS, Loriot Y, Rosenberg JE, Fong L, Mellman I, Chen DS, Green M, Derleth C, Fine GD, Hegde PS, Bourgon R, Powles T (2018). TGFβ attenuates tumour response to PD-L1 blockade by contributing to exclusion of T cells. Nature.

[50] Duan X, Wang W, Pan Q, Guo L (2016). Type 2 diabetes mellitus intersects with pancreatic cancer diagnosis and development. Front Oncol.

[51] Reni M, Dugnani E, Cereda S, Belli C, Balzano G, Nicoletti R, Liberati D, Pasquale V, Scavini M, Maggiora P, Sordi V, Lampasona V, Ceraulo D, Di Terlizzi G, Doglioni C, Falconi M, Piemonti L (2016). (Ir)relevance of metformin treatment in patients with metastatic pancreatic cancer: an open-label, randomized Phase II Trial. Clin Cancer Res.

[52] García Rubiño ME, Carrillo E, Ruiz Alcalá G, Domínguez-Martín A, Marchal A, Boulaiz H (2019). Phenformin as an anticancer agent: challenges and prospects. Int J Mol Sci..

[53] Monda KL, Chen GK, Taylor KC (2013). A meta-analysis identifies new loci associated with body mass index in individuals of African ancestry. Nat Genet.

[54] Liu H, Wang L, Guo Z, Xu Q, Fan W, Xu Y, Hu J, Zhang Y, Tang J, Xie M, Zhou Z, Hou S (2021). Genome-wide association and selective sweep analyses reveal genetic loci for FCR of egg production traits in ducks. Genet Sel Evol.

[55] Dhanoa BS, Cogliati T, Satish AG, Bruford EA, Friedman JS (2013). Update on the Kelch-like (KLHL) gene family. Hum Genomics.

[56] Furukawa M, He YJ, Borchers C, Xiong Y (2003). Targeting of protein ubiquitination by BTB-Cullin 3-Roc1 ubiquitin ligases. Nat Cell Biol.

[57] Xu L, Wei Y, Reboul J, Vaglio P, Shin TH, Vidal M, Elledge SJ, Harper JW (2003). BTB proteins are substrate-specific adaptors in an SCF-like modular ubiquitin ligase containing CUL-3. Nature.

[58] Schwenk RW, Vogel H, Schürmann A (2013). Genetic and epigenetic control of metabolic health. Mol Metab.

