# Supplementary material for: Identification of stromal microenvironment characteristics and key molecular mining in pancreatic cancer
Source: Discov Oncol. 2022 Aug 25;13:83. doi: 10.1007/s12672-022-00532-y (PMC9411435; doi:10.1007/s12672-022-00532-y)
Supplement: Supplementary file 1 — (DOCX 6151 KB) Fig. S1 Flowchart of this study. Fig. S2 ROC curve of the Lasso model (Coefficient 1 was obtained at the minimum MSE, and coefficient 2 was obtained at 1 SE MSE. Cutoff values were obtained based on the Youden index). Fig. S3 GO enrichment analysis between PECMS groups. Fig. S4 Level of angiogenesis marker genes and pathway GSVA scores between PECMS groups (ns: no significant difference; *: P<0.05; **: P<0.005; ***: P<0.0005; ****: P<0.00005). Fig. S5 CIBERSORT score value of different infiltrating immune cells between PECMS groups. Fig. S6 Predictive value of molecular targeted drugs between PECMS groups (ns: no significant difference; *: P<0.05; **: P<0.005; ***: P<0.0005; ****: P<0.00005). Fig. S7 Survival curve of TCGA-PAAD. The patients were divided by the median value of PECMS feature gene mRNA levels. Fig. S8 Validation of disease-free survival, immunological characteristics, and drug sensitivity in our retrospective data set (a: Disease-free survival of different PECMS groups. b: The normalized mRNA expression of immune feature genes. c: The normalized pathway GSVA scores in different PECMS groups. d: Drug sensitivity prediction of chemotherapy drugs in different PECMS groups (L: PECMS-low; H: PECMS-high) and correlation between PECMS and drug sensitivity prediction). Fig. S9 IHC of PD-L1, CD8, CD31, and GLUT1 (SLC2A1) in our single-center retrospective cohort (a: IHC staining, the patients were grouped by the level of KLHL32. b: Positive and negative controls of the 4 antibodies. CD31: human lung tissue; GLUT1: rat liver tissue; CD8: rat spleen tissue; PD-L1: rat heart tissue) [file 12672_2022_532_MOESM1_ESM.docx]

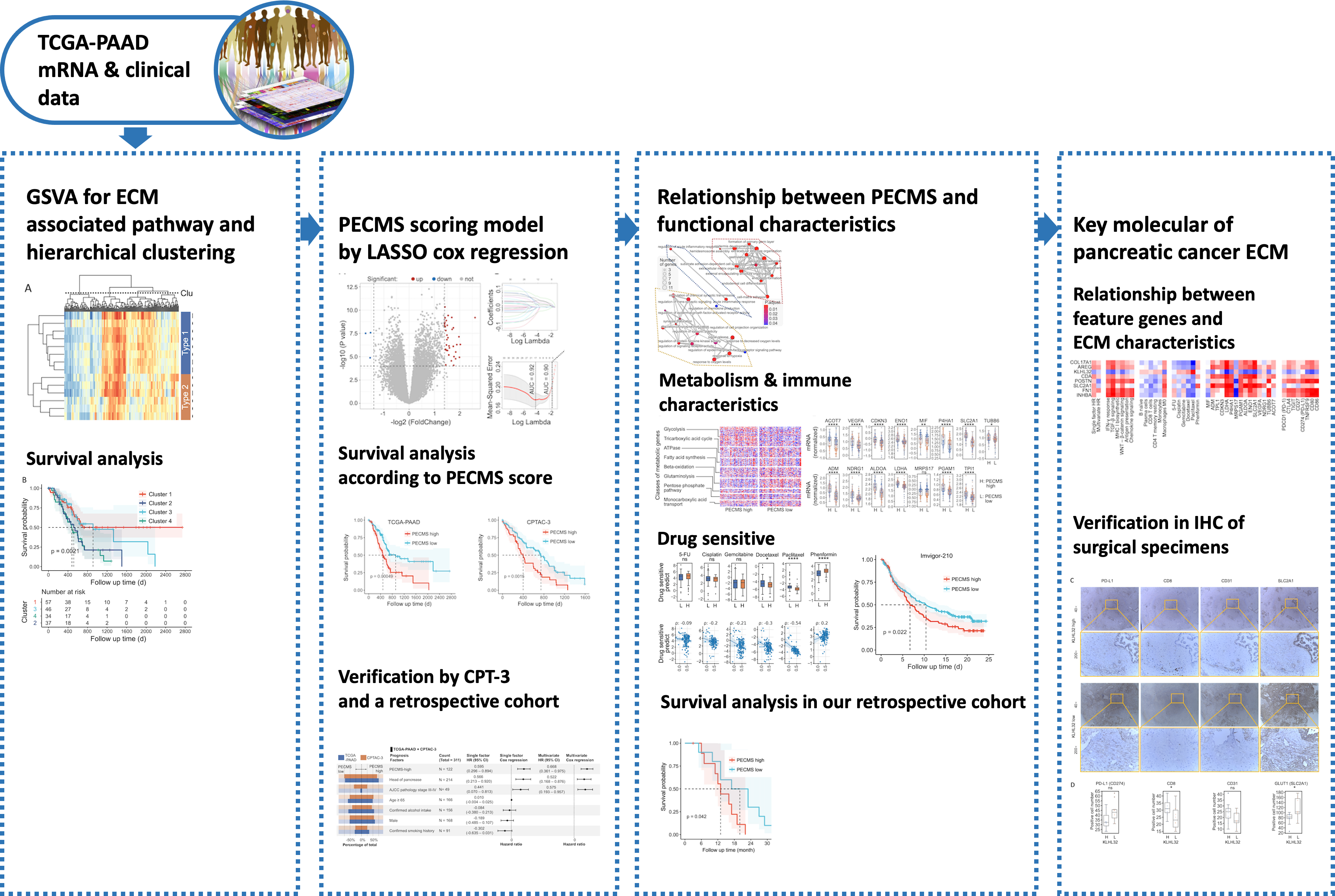


**Supplementary Fig. S1** Flowchart of this study


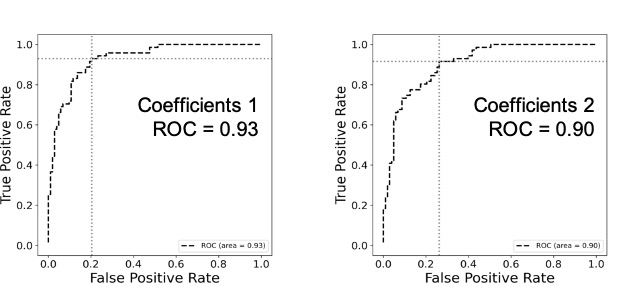


**Supplementary Fig. S2** ROC curve of the Lasso model (Coefficient 1 was obtained at the minimum MSE, and coefficient 2 was obtained at 1 SE MSE. Cutoff values were obtained based on the Youden index)


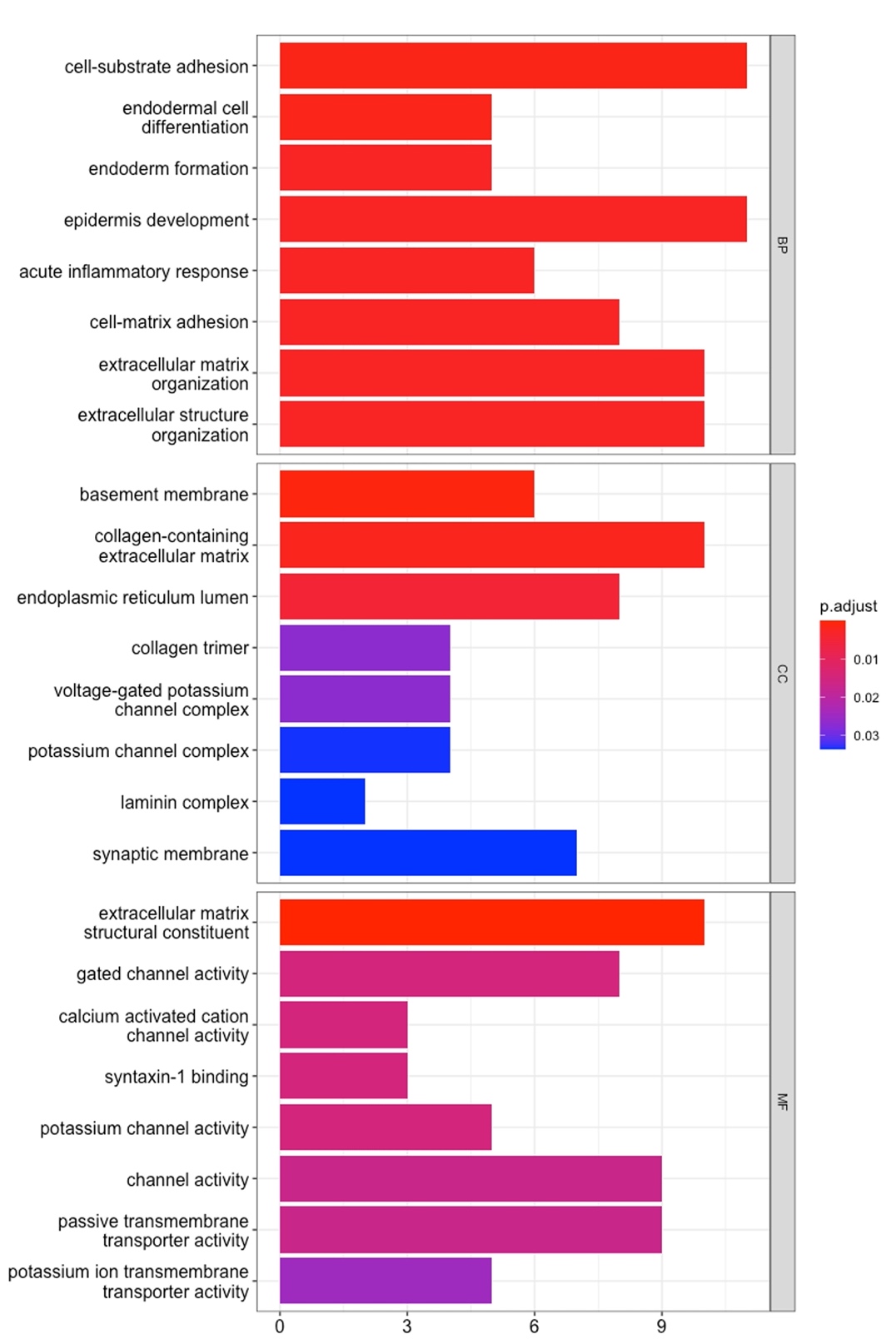


**Supplementary Fig. S3** GO enrichment analysis between PECMS groups


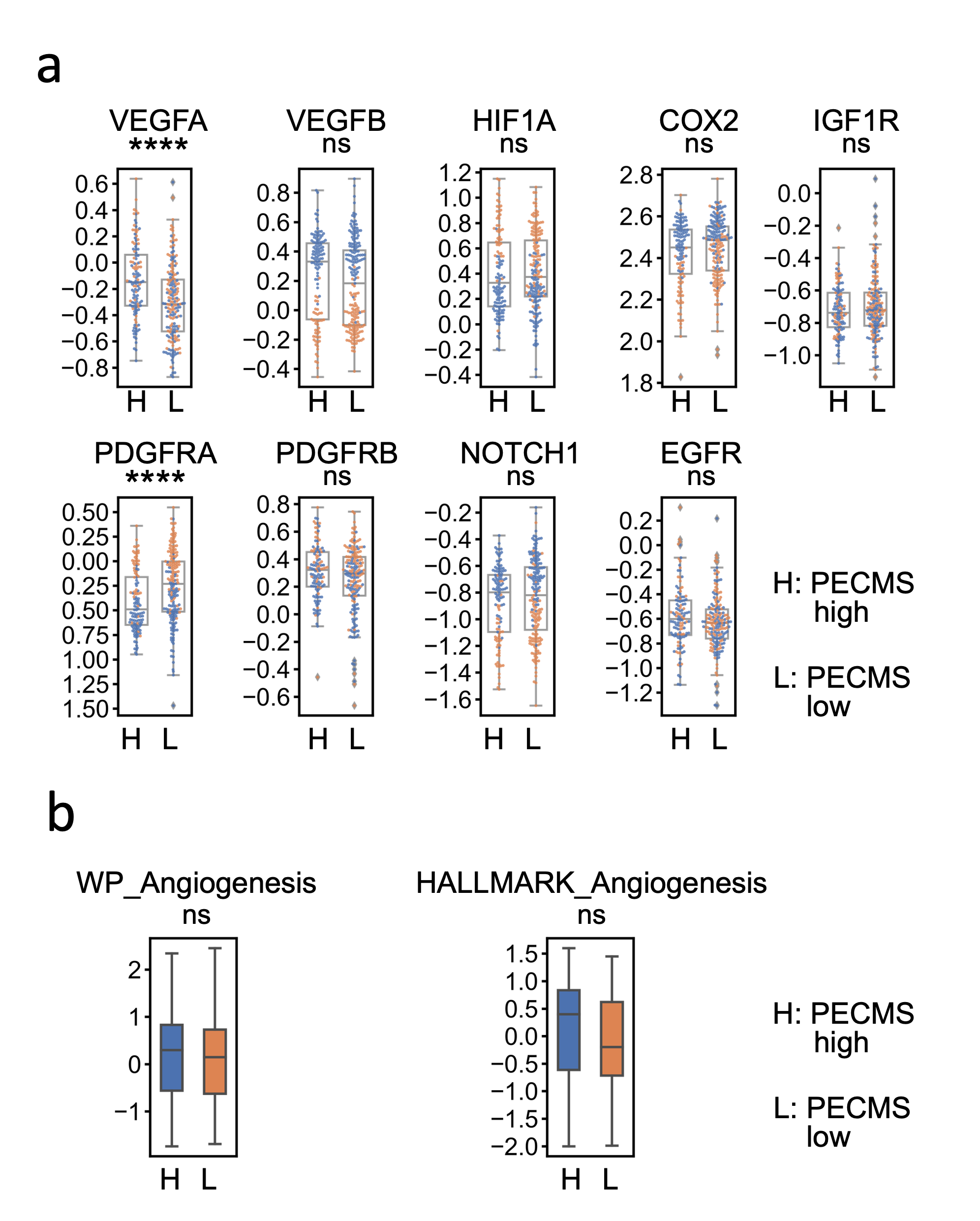


**Supplementary Fig. S4** Level of angiogenesis marker genes and pathway GSVA scores between PECMS groups (ns: no significant difference; *: P<0.05; **: P<0.005; ***: P<0.0005; ****: P<0.00005)


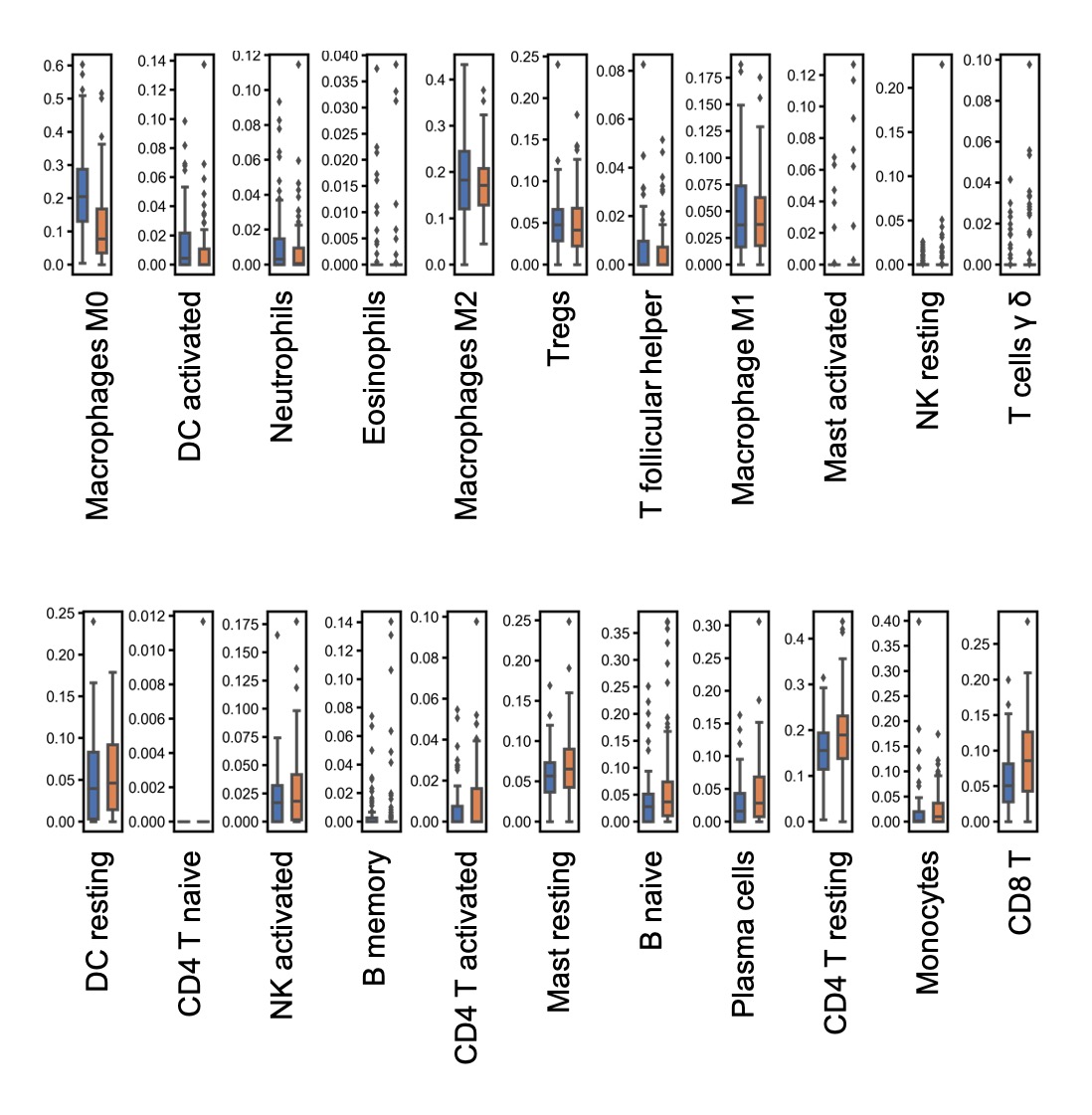


**Supplementary Fig. S5** CIBERSORT score value of different infiltrating immune cells between PECMS groups


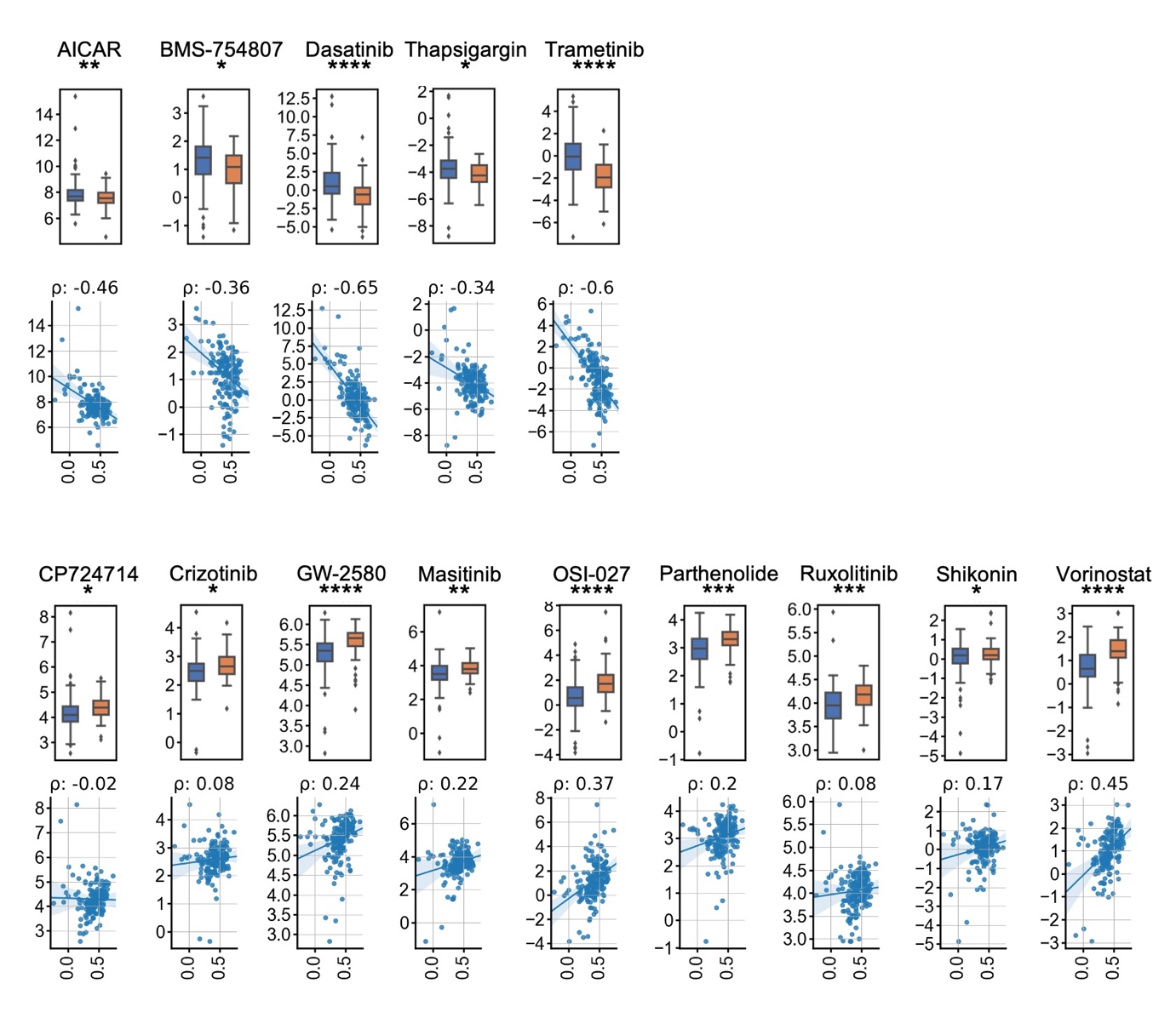


**Supplementary Fig. S6** Predictive value of molecular targeted drugs between PECMS groups (ns: no significant difference; *: P<0.05; **: P<0.005; ***: P<0.0005; ****: P<0.00005)


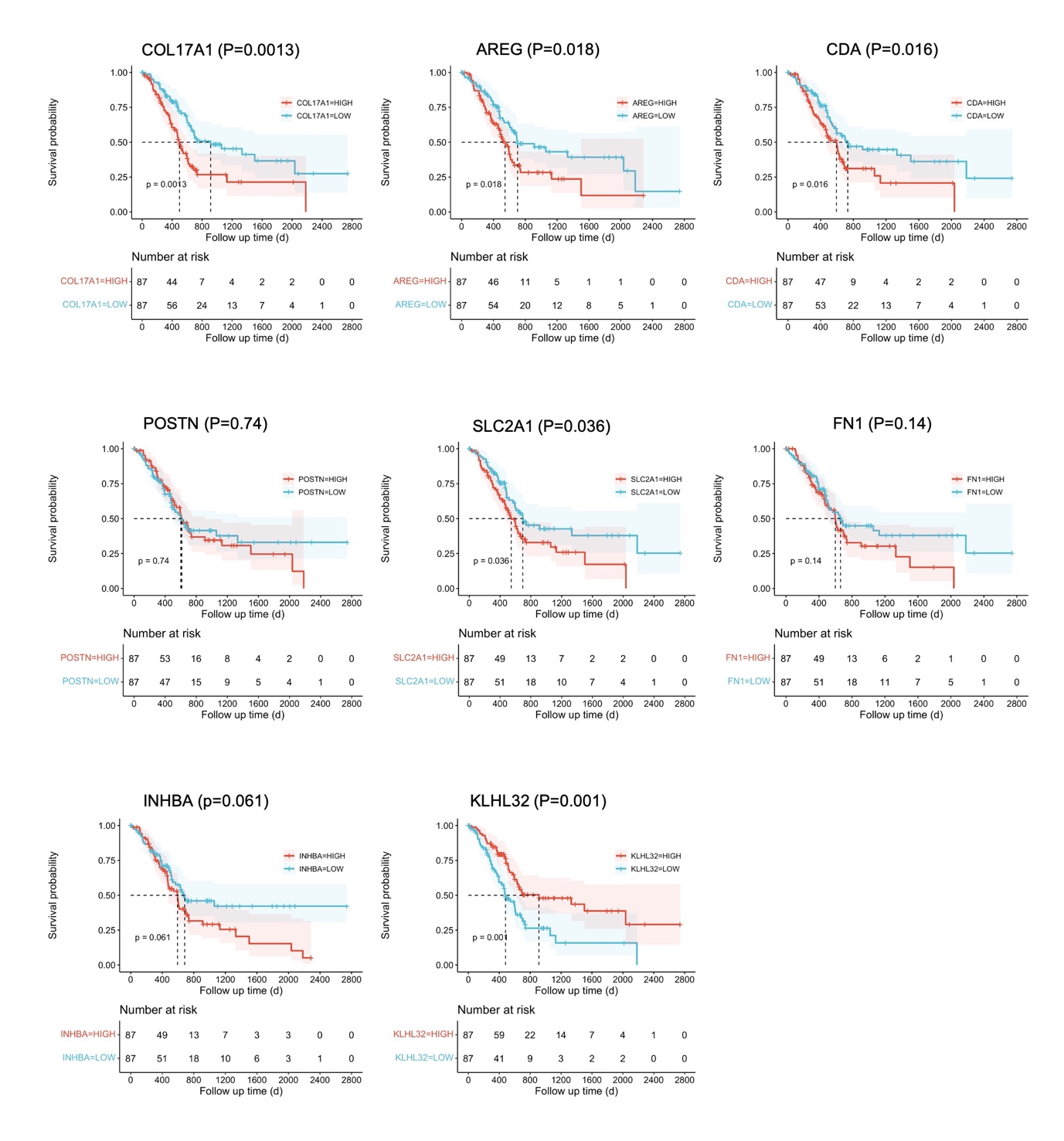


**Supplementary Fig. S7** Survival curve of TCGA-PAAD. The patients were divided by the median value of PECMS feature gene mRNA levels


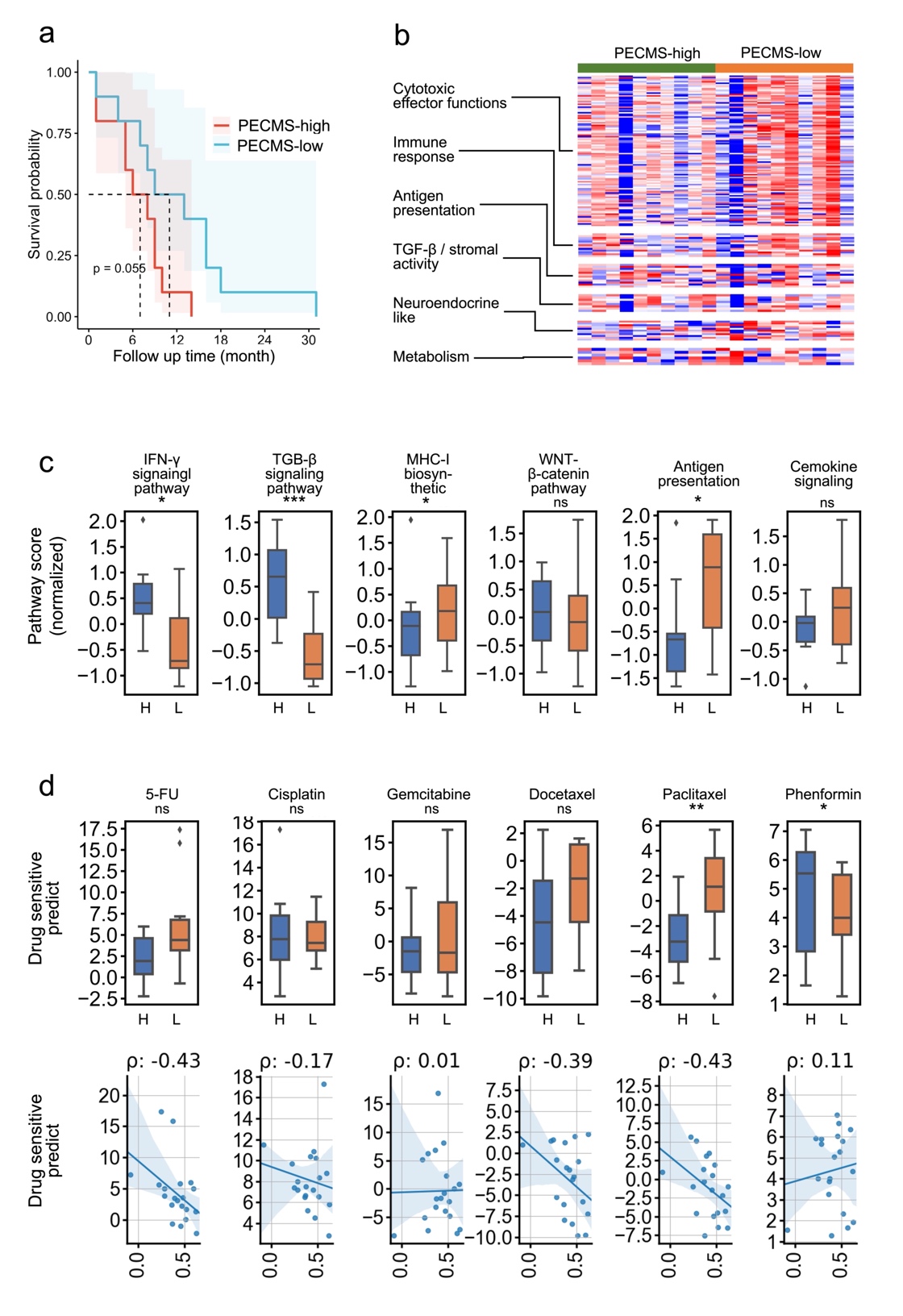


**Supplementary Fig. S8** Validation of disease-free survival, immunological characteristics, and drug sensitivity in our retrospective data set (a: Disease-free survival of different PECMS groups. b: The normalized mRNA expression of immune feature genes. c: The normalized pathway GSVA scores in different PECMS groups. d: Drug sensitivity prediction of chemotherapy drugs in different PECMS groups (L: PECMS-low; H: PECMS-high) and correlation between PECMS and drug sensitivity prediction)


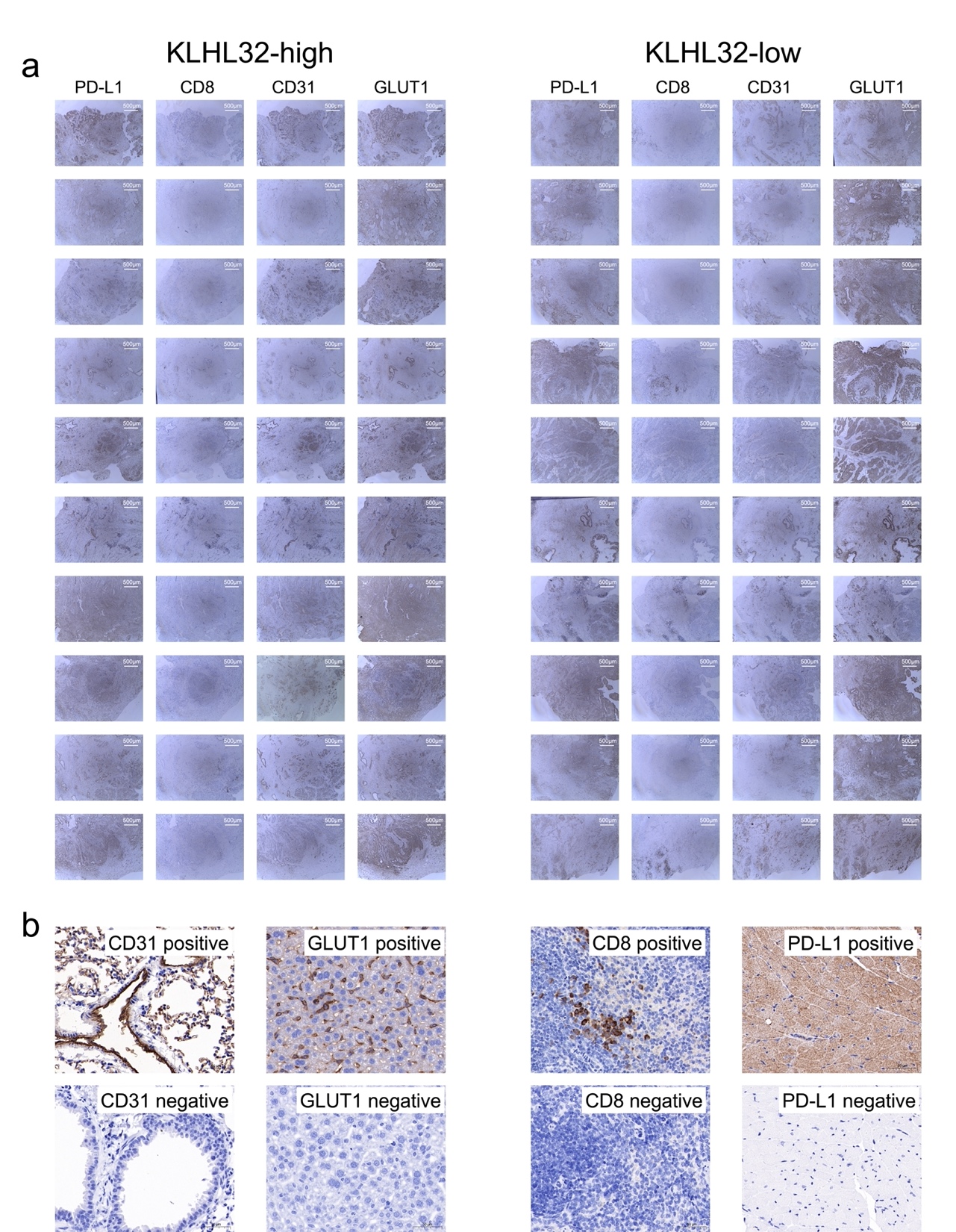


**Supplementary Fig. S9** IHC of PD-L1, CD8, CD31, and GLUT1 (SLC2A1) in our single-center retrospective cohort (a: IHC staining, the patients were grouped by the level of KLHL32. b: Positive and negative controls of the 4 antibodies. CD31: human lung tissue; GLUT1: rat liver tissue; CD8: rat spleen tissue; PD-L1: rat heart tissue)
